# Supplementary material for: Identifying modifiable risk factors for mortality in children aged 1–59 months admitted with WHO-defined severe pneumonia: a single-centre observational cohort study from rural Malawi
Source: BMJ Paediatr Open. 2022 Apr 19;6(1):e001330. doi: 10.1136/bmjpo-2021-001330 (PMC9020281; doi:10.1136/bmjpo-2021-001330)
Supplement: Supplementary data [file bmjpo-2021-001330supp001.pdf]

Supplemental Materials

Supplemental Table 1: Sensitivity analysis of survival and death, excluding children with no malaria result

Factor		Survived	Died	p-value
N=877		856	21	
Sex, n (%)	Males	505 (59.0%)	12 (57.1%)	1.00
	Females	351 (41.0%)	9 (42.9%)	
Age in months, median (IQR)		9.2 (4.6, 17.2)	8.1 (6.5, 12.0)	0.76
Respiratory rate (breaths/min), mean (SD)		59.6 (12.9)	60.3 (13.7)	0.82
Fast breathing for age	No	143 (16.7%)	3 (14.3%)	1.00
	Yes	713 (83.3%)	18 (85.7%)	
Pulse rate (beats/min), mean (SD)		162.3 (20.4)	170.0 (30.4)	0.09
Pulse rate >180 beats/min, n (%)	No	397 (77.8%)	10 (62.5%)	0.22
	Yes	113 (22.2%)	6 (37.5%)	
Pulse rate >160 beats/min, n (%)	No	181 (52.5%)	1 (20.0%)	0.20
	Yes	164 (47.5%)	4 (80.0%)	
Severe chest indrawing, n (%)	No	99 (11.6%)	4 (19.0%)	0.30
	Yes	757 (88.4%)	17 (81.0%)	
Head nodding, n (%)	No	676 (79.0%)	13 (61.9%)	0.10
	Yes	180 (21.0%)	8 (38.1%)	
Grunting, n (%)	No	754 (88.2%)	11 (52.4%)	<0.01
	Yes	101 (11.8%)	10 (47.6%)	
Stridor when calm, n (%)	No	844 (98.6%)	19 (90.5%)	0.04
	Yes	12 (1.4%)	2 (9.5%)	
Apnea, n (%)	No	850 (99.3%)	20 (95.2%)	0.16
	Yes	6 (0.7%)	1 (4.8%)	
Nasal flaring, n (%)	No	350 (40.9%)	9 (42.9%)	1.00
	Yes	506 (59.1%)	12 (57.1%)	
MUAC in cm, mean (SD)		14.0 (1.2)	13.3 (1.0)	<0.01
MUAC 11.5-13.5 cm, n (%)	No	604 (70.6%)	11 (52.4%)	0.09
	Yes	252 (29.4%)	10 (47.6%)	
Hb (g/dL), mean (SD)		10.0 (2.0)	7.8 (3.0)	<0.01
Hb < 5 g/dL	No	832 (97.2%)	18 (85.7%)	0.02
	Yes	24 (2.8%)	3 (14.3%)	
SpO2 (%), median (IQR)		96.0 (95.0, 98.0)	96.0 (94.0, 98.0)	0.49

SpO2 93-100%	0	781 (91.2%)	18 (85.7%)	0.42
SpO2 90-92%	1	75 (8.8%)	3 (14.3%)	
Malaria positive, n (%)	No	663 (77.5%)	10 (47.6%)	0.003
	Yes	193 (22.5%)	11 (52.4%)	

Supplemental Table 2. Characteristics of children 1-59 months old with WHO-defined severe pneumonia at Salima District Hospital, Malawi in an observational study compared to a randomized controlled trial CPAP IMPACT

Characteristic	Observational study N=884	CPAP IMPACT N=644	p-value
Age in months, median (IQR)	9.2 (4.7, 17.2)	7.7 (3.1, 15.2)	<0.01
Females, n (%)	362 (41.0%)	299 (46.4%)	0.03
Males, n (%)	522 (59.0%)	345 (53.6%)	
Weight in kg, mean (SD)	8.5 (2.5)	7.2 (2.8)	<0.01
MUAC in cm, mean (SD)	14.0 (1.2)	12.7 (1.9)	<0.01
HIV-infected, n (%)	0 (0.0%)	36 (5.6%)	<0.01
HIV-exposed uninfected, n (%)	0 (0.0%)	99 (15.4%)	<0.01
Severe malnutrition, n (%)	0 (0.0%)	222 (34.5%)	<0.01
Hypoxemia only, n (%)	0 (0.0%)	287 (44.6%)	<0.01
Pulse rate in beats/min, mean (SD)	162.4 (20.7)	164.1 (24.0)	0.15
Respiratory rate in breaths/min, mean (SD)	59.6 (12.9)	61.9 (15.1)	<0.01
SpO_2, median (IQR)	96 (95, 98)	88 (83, 94)	<0.01
SpO_2 93-100%, n (%)	805 (91.1%)	191 (29.7%)	
SpO_2 90-92%, n (%)	79 (8.9%)	38 (5.9%)	
SpO_2 <90%, n (%)	0 (0.0%)	415 (64.4%)	
1 or more respiratory danger signs[1], n (%)	866 (98.0%)	638 (99.1%)	0.09
Severe chest indrawing, n (%)	777 (87.9%)	575 (89.3%)	0.41
Head nodding, n (%)	188 (21.3%)	176 (27.3%)	<0.01
Grunting, n (%)	112 (12.7%)	154 (23.9%)	<0.01
Very fast breathing for age, n (%)[2]	256 (29.0%)	211 (32.8%)	0.11
Stridor when calm, n (%)	14 (1.6%)	13 (2.0%)	0.52
Apnea, n (%)	7 (0.8%)	37 (5.7%)	<0.01
Nasal flaring, n (%)	519 (58.7%)	471 (73.1%)	<0.01
Convulsions, n (%)	45 (5.1%)	61 (9.5%)	<0.01
Inability to feed, n (%)	27 (3.1%)	78 (12.1%)	<0.01
Vomiting everything, n (%)	12 (1.4%)	19 (3.0%)	0.03
Blantyre Coma Score ≤4, n (%)	20 (2.3%)	55 (8.5%)	<0.01
Crackles on auscultation, n (%)	429 (48.5%)	311 (48.3%)	0.95
Wheeze on auscultation, n (%)	160 (18.1%)	128 (19.9%)	0.38
Hb <5 g/dL, n (%)	27 (3.1%)	50 (7.8%)	<0.01
Hb (g/dL), mean (SD)	10.0 (2.1)	9.5 (2.6)	<0.01
Malaria positive, n (%)[3]	204 (23.3%)	197 (30.6%)	<0.01
Death, n (%)	21 (2.4%)	88 (13.7%)	<0.01

WHO indicates World Health Organization; IQR, interquartile range; SD, standard deviation; MUAC, mid-upper arm circumference; SpO_2; peripheral oxyhemoglobin saturation; Hb, hemoglobin.

[1]Any of the following signs of respiratory distress: severe chest indrawing, head nodding, grunting, very fast breathing for age, stridor when calm, apnea, nasal flaring

[2]≥80 breaths/min if 30-59 days old, ≥70 breaths/min if 2-11 months old, and ≥60 breaths/min if 12-59 months old
[3]Rapid test positive, 10 observational study participants were missing test results
