# Supplementary material for: Identifying modifiable risk factors for mortality in children aged 1–59 months admitted with WHO-defined severe pneumonia: a single-centre observational cohort study from rural Malawi
Source: BMJ Paediatr Open. 2022 Apr 19;6(1):e001330. doi: 10.1136/bmjpo-2021-001330 (PMC9020281; doi:10.1136/bmjpo-2021-001330)
Supplement: Author's manuscript [file bmjpo-2021-001330.draft_revisions.pdf]

BMJ Paediatrics Open

BMJ Paediatrics Open is committed to open peer review. As part of this commitment we make the peer review history of every article we publish publicly available.

When an article is published we post the peer reviewers' comments and the authors' responses online. We also post the versions of the paper that were used during peer review. These are the versions that the peer review comments apply to.

The versions of the paper that follow are the versions that were submitted during the peer review process. They are not the versions of record or the final published versions. They should not be cited or distributed as the published version of this manuscript.

BMJ Paediatrics Open is an open access journal and the full, final, typeset and author-corrected version of record of the manuscript is available on our site with no access controls, subscription charges or pay-per-view fees (http://bmjpaedsopen.bmj.com).

If you have any questions on BMJ Paediatrics Open's open peer review process please email info.bmjpo@bmj.com

BMJ Paediatrics Open

# World Health Organization-defined severe pneumonia among hospitalized children without HIV, severe malnutrition, and hypoxemia in rural Malawi: a single center prospective observational study

| | |
|---|---|
| Journal: | *BMJ Paediatrics Open* |
| Manuscript ID | bmjpo-2021-001330 |
| Article Type: | Original research |
| Date Submitted by the Author: | 25-Oct-2021 |
| Complete List of Authors: | Eckerle, Michelle; Cincinnati Children's Hospital Medical Center, Pediatrics<br>Mvalo, Tisungane; University of North Carolina Project; University of North Carolina at Chapel Hill School of Medicine, Department of Pediatrics<br>Smith, Andrew; The University of Utah School of Medicine<br>Kondowe, Davie; University of North Carolina Project<br>Makonokaya, Don; University of North Carolina Project<br>Vaidya, Dhananjay; Johns Hopkins University School of Medicine<br>Hosseinipour, Mina; University of North Carolina Project; University of North Carolina at Chapel Hill School of Medicine<br>McCollum, Eric; Johns Hopkins School of Medicine, Department of Paediatrics |
| Keywords: | Epidemiology, Health services research, Mortality |
| | |

SCHOLARONE™
Manuscripts

*I, the Submitting Author has the right to grant and does grant on behalf of all authors of the Work (as defined in the below author licence), an exclusive licence and/or a non-exclusive licence for contributions from authors who are: i) UK Crown employees; ii) where BMJ has agreed a CC-BY licence shall apply, and/or iii) in accordance with the terms applicable for US Federal Government officers or employees acting as part of their official duties; on a worldwide, perpetual, irrevocable, royalty-free basis to BMJ Publishing Group Ltd ("BMJ") its licensees and where the relevant Journal is co-owned by BMJ to the co-owners of the Journal, to publish the Work in this journal and any other BMJ products and to exploit all rights, as set out in our licence.*

*The Submitting Author accepts and understands that any supply made under these terms is made by BMJ to the Submitting Author unless you are acting as an employee on behalf of your employer or a postgraduate student of an affiliated institution which is paying any applicable article publishing charge ("APC") for Open Access articles. Where the Submitting Author wishes to make the Work available on an Open Access basis (and intends to pay the relevant APC), the terms of reuse of such Open Access shall be governed by a Creative Commons licence – details of these licences and which Creative Commons licence will apply to this Work are set out in our licence referred to above.*

*Other than as permitted in any relevant BMJ Author's Self Archiving Policies, I confirm this Work has not been accepted for publication elsewhere, is not being considered for publication elsewhere and does not duplicate material already published. I confirm all authors consent to publication of this Work and authorise the granting of this licence.*

Confidential: For Review Only

# World Health Organization-defined severe pneumonia among hospitalized children without HIV, severe malnutrition, and hypoxemia in rural Malawi: a single center prospective observational study

Michelle Eckerle, MD[1,2]

Tisungane Mvalo, FC Paed[3,4]

Andrew G. Smith, MD[5]

Davie Kondowe, BBiomedSc[3]

Don Makonokaya, BScPH[3]

Dhananjay Vaidya, PhD[6]

Mina C. Hosseinipour, MD[3,8]

Eric D. McCollum, MD, MPH[9,10]

[1]Division of Emergency Medicine, Cincinnati Children's Hospital Medical Center, Cincinnati, Ohio, USA

[2]Department of Pediatrics, University of Cincinnati College of Medicine, Cincinnati, Ohio, USA

[3]University of North Carolina Project Malawi, Lilongwe, Malawi

[4]Department of Pediatrics, School of Medicine, University of North Carolina at Chapel Hill, NC, USA.

[5]Department of Pediatrics, University of Utah School of Medicine, Salt Lake City, Utah, USA

Confidential: For Review Only

[6]Department of Medicine, Epidemiology and the BEAD Core, Johns Hopkins University, Baltimore, MD, USA

[7]Department of Pediatrics, BEAD Core, Johns Hopkins University, Baltimore, MD, USA

[8]Division of Infectious Disease, University of North Carolina at Chapel Hill, Chapel Hill, North Carolina, USA

[9]Global Program for Respiratory Sciences, Eudowood Division of Pediatric Respiratory Sciences, Department of Pediatrics, School of Medicine, Johns Hopkins University, Baltimore, Maryland, United States

[10]Department of International Health, Bloomberg School of Public Health, Johns Hopkins University, Baltimore, Maryland, United States

Corresponding author:

Eric D. McCollum, MD, MPH

Email: emccoll3@jhmi.edu

Word count: 2,488

Confidential: For Review Only

Abstract:

Objective: Although HIV-infection, severe malnutrition, and hypoxemia are associated with high mortality in children with World Health Organization (WHO)-defined severe pneumonia in sub-Saharan Africa, many do not have these conditions and yet mortality remains elevated. Further stratifying mortality risk for children without these conditions could permit more strategic resource utilization and more optimal outcomes. We therefore evaluated associations between mortality and clinical characteristics not currently recognized by the WHO as high-risk among children in Malawi with severe pneumonia but without HIV (including exposure), severe malnutrition, and hypoxemia.

Methods: Between May 2016 and March 2018 we conducted a prospective observational study alongside a randomized controlled trial (CPAP IMPACT) at Salima District Hospital in Salima, Malawi. Hospitalized 1-59 month olds with WHO-defined severe pneumonia without severe malnutrition, HIV, and/or hypoxemia were enrolled. CPAP IMPACT participants differed in that all had severe malnutrition, HIV, and/or hypoxemia. Study staff assessed children at admission and ascertained hospital outcomes. We compared group characteristics using Student t, rank sum, Chi-Square or Fisher's Exact tests as appropriate.

Results: Observational study participants (N=884) were older (median 9.2 months vs 7.7 months), more frequently male (59.0% vs 53.5%), and had lower mortality (2.4% vs 13.7%) than trial participants (N=643). Among 884 observational participants grunting (10/112 (8.9%) vs 11/772 (1.4%)), stridor (2/14 (14.2%) vs 19/870 (2.1%)), apnea (1/7

Confidential: For Review Only

(14.2%) vs 20/877 (2.2%)), hemoglobin <5 g/dL (3/27 (11.1%) vs 18/857 (2.1%)), and malaria (11/204 (5.3%) vs 10/680 (1.4%)) were associated with mortality compared to children without these characteristics. Observational study survivors had a 2.2 g/dL higher mean hemoglobin and 0.7 cm higher mean arm circumference than those who died.

Conclusion: In this single-center study, our analysis identifies potentially modifiable clinical features exist that could further stratify mortality risk among hospitalized Malawian children with severe pneumonia lacking recognized high-risk conditions.

Confidential: For Review Only

What is known about this subject

- Lower respiratory infections like pneumonia kill an estimated 800,000 children annually, making it the number one infectious cause of pediatric deaths globally.
- Among children with pneumonia, important drivers of mortality in low-income and middle-income countries (LMICs) are severe malnutrition, HIV infection or exposure, and hypoxemia.
- Children in LMICs with severe pneumonia but without HIV (including exposure), severe malnutrition, and hypoxemia account for a large proportion of the overall pneumonia burden and mortality.

What this study adds

- Detailed description of rural, hospitalized Malawian children with pneumonia but without severe malnutrition, HIV infection or exposure, and hypoxemia in the post pneumococcal and *Haemophilus influenzae* vaccine era.
- An examination of the association between clinical characteristics and mortality among Malawian children hospitalized with severe pneumonia and lacking severe malnutrition, HIV, and hypoxemia.
- Recommendations and next steps for translating research evidence into policy guidance to benefit African children with severe pneumonia.

Confidential: For Review Only

Introduction:

Pneumonia is a leading cause of death among children under 5 years worldwide and this mortality burden is disproportionately borne by children in low-income and middle-income countries (LMICs).[1] Severe malnutrition, HIV-infection or exposure, and hypoxemia (peripheral oxyhemoglobin saturation ($SpO_2$) <90%) are known to place children with pneumonia at higher risk of death.[1-4] While children with severe pneumonia but without these conditions are at relatively lower mortality risk, they still comprise a substantial overall burden of pneumonia morbidity and mortality in LMICs.[1] Identifying unique clinical characteristics that both predict mortality and are modifiable among pediatric pneumonia cases lacking severe malnutrition, HIV (including exposure), and hypoxemia may represent an effective strategy for lowering pneumonia deaths in LMICs.

The World Health Organization (WHO) recommends a syndromic approach to child pneumonia diagnosis and management in LMICs to improve implementation feasibility.[5] Recommended treatment for WHO-defined severe pneumonia includes hospital admission, parenteral antibiotics, and supplemental oxygen.[5] Understanding which children with WHO-defined severe pneumonia in LMICs are at high risk of death is important as it can impact resource allocation in austere settings where advanced respiratory support and other resources are limited.[6]

In conjunction with a randomized controlled trial called CPAP IMPACT, which studied children with WHO-defined severe pneumonia and at least one high-risk condition



Confidential: For Review Only

(severe malnutrition, HIV-infection or exposure, and/or hypoxemia),[7,8] we conducted a parallel prospective observational study of children with WHO-defined severe pneumonia but without any of these three high-risk conditions. Our objectives were to contextualize these children by comparing them to trial participants with these conditions and then to identify predictors of mortality within this observational group.

Methods:

We conducted a pre-specified sub-study in conjunction with a randomized controlled trial called CPAP IMPACT.[7,8] CPAP IMPACT compared bubble continuous positive airway pressure (bCPAP) to low flow oxygen for children with WHO-defined severe pneumonia and at least one high-risk condition. The trial took place at Salima District Hospital in Salima, Malawi, which is a secondary hospital lacking intensive care resources or portable chest radiography, from June 2015 through March 2018. From May 2016 through March 2018, children with WHO-defined severe pneumonia were invited to participate in this prospective observational study if ineligible for CPAP IMPACT. The protocol and results for the trial were published previously.[7,8] Institutional review boards approved the protocol. Enrolled participants provided written consent.

Children admitted to Salima District Hospital were screened for eligibility seven days per week and at all times during the study. Children were eligible for CPAP IMPACT if 1-59 months with WHO-defined severe pneumonia and HIV-infection or exposure, severe malnutrition (weight for age z score < -3 standard deviations (SD), mid-upper arm circumference (MUAC) <11.5 cm, and/or bilateral pedal edema), or a $SpO_2$ <90%.



Confidential: For Review Only

Children without a high-risk condition but with severe pneumonia and 1-59 months were eligible for this observational study.

Per WHO guidelines, children could meet severe pneumonia criteria by having cough and/or difficult breathing plus signs of pneumonia with at least one general danger sign or signs of respiratory distress with or without a general danger sign.[9] Respiratory distress included head nodding, nasal flaring, grunting, stridor at rest, tracheal tugging, severe chest indrawing, very fast breathing for age (≥80 breaths/min if 30-59 days old, ≥70 breaths/min if 2-11 months old, and ≥60 breaths/min if 12-59 months old), or apnea. Signs of pneumonia included fast breathing (60-79 breaths/min if 30-59 days old, 50-69 breaths/min if 2-11 months old, and 40-59 breaths/min if 12-59 months old) and/or chest wall indrawing. Inability to feed, vomiting everything, lethargic or unconscious (Blantyre Coma Score ≤4), and convulsions comprised general danger signs.

After enrollment, participants were assessed for malaria (SD Bioline (HRP2/pLDH) rapid antigen tests (Abbott, Johannesburg, South Africa)) and anemia (Hemocue 301+). For observational participants study staff recommended initial evaluations and treatments but thereafter children received usual care per local protocols administered by government hospital staff, including oxygen, antibiotics, and fluids. Children in the observational study were followed passively by study staff until hospital outcome. This study's primary outcome was in-hospital mortality.

Confidential: For Review Only

*Statistical Analysis*

Bivariable analysis comparing clinical variables with in-hospital mortality was performed as follows: The Student's *t*-test was used to assess differences between the means of normally distributed continuous variables (tabulated as means and standard deviations (SDs)), the non-parametric Mann-Whitney U test evaluated differences in the rank sums of continuous variables (tabulated as medians and interquartile range (IQR) presented as 25$^{th}$ and 75$^{th}$ percentile, Chi Square and Fisher's Exact tests (if the large sample assumption could not be justified) analyzed categorical clinical variables (tabulated as numbers and percentages). Although we planned to fit multivariable regression models we determined that it was not advisable due to the relatively limited number of mortality events experienced by observational study participants. All analyses were conducted using Stata (version 15.1; StataCorp, College Station, Texas).

Patient and public involvement

Patients and the public in Malawi were sensitized to the development, design, recruitment and conduct via local meetings before, during, and after the research.

Results:

From June 2015 through March 2018, 1,712 children were screened and 644 met inclusion criteria for CPAP IMPACT (Figure 1). From May 2016 enrollment began for the observational study and concluded at the same time as the parent trial in March 2018. Among 1,062 children that did not meet inclusion criteria for CPAP IMPACT, 884 met criteria for the observational study and agreed to participate.



Confidential: For Review Only

We compared observational participants to children enrolled in CPAP IMPACT to provide context for both groups and the spectrum of pneumonia at Salima District Hospital during the study period (Table 1). Observational participants did not have severe malnutrition, HIV, or hypoxemia. Patients in the observational study, compared to those in the trial, were 1.5 months older (median 9.2 months [IQR 4.7, 17.2] vs median 7.7 months [IQR 3.1, 15.2], rank sum $p<0.01$) and 5.4% more were male (59.0% vs 53.6%, $p=0.03$). Observational participants were less severely ill than trial participants, as exemplified by their 2.3 breaths/minute lower mean respiratory rate (59.6 breaths/min [SD 12.9] vs 61.9 breaths/min [SD 15.1], $p<0.01$), 8% higher $SpO_2$ (median 96% [IQR 95, 98] vs median 88% [IQR 83, 94], rank sum $p<0.01$), and lower proportion with signs of severe respiratory distress including head nodding (21.3% vs 27.3%, $p<0.01$), grunting (12.7% vs 23.9%, $p<0.01$), apnea (0.8% vs 5.7%, $p<0.01$), and nasal flaring (58.7% vs 73.1%, $p<0.01$). In addition, all WHO-defined general danger signs including convulsions (5.1% vs 9.5%, $p<0.01$), inability to feed (3.1% vs 12.1%, $p<0.01$), vomiting everything (1.4% vs 3.0%, $p=0.03$), and an altered mental status as measured by the Blantyre Coma Score (2.3% vs 8.5%, $p<0.01$), were less frequent among observational than CPAP IMPACT children. Compared to children in the trial, a 2.5 fold lower proportion of observational children had a hemoglobin <5 g/dL (3.1% vs 7.9%, $p<0.01$). Observational children also had a 0.5 g/dL higher mean hemoglobin (10.0 g/dL [SD 2.1] vs 9.5 g/dL [SD 2.6], $p<0.01$) and a lower proportion had a positive malaria test (23.3% vs 30.06%, $p<0.01$) than in CPAP IMPACT. Perhaps most importantly, the mortality of observational children was 11.3 absolute percentage



Confidential: For Review Only

points lower than those in the trial (2.4% vs 13.7%, p<0.01). Altogether, when accounting for trial participants and the observational group, the hospital mortality of severe pneumonia during the study period was 7.1% (109/1,528).

Given that our observational group represented a cohort of lower acuity children with severe pneumonia than those in the trial, we sought to identify whether any observational participant characteristics – with an emphasis on characteristics not included in the WHO IMCI child pneumonia guidelines[5] – had associations with hospital mortality (Table 2). Among observational participants respiratory distress signs like grunting (10/112 (8.9%) vs 11/772 (1.4%), p<0.01), stridor at rest (2/14 (14.2%) vs 19/870 (2.1%), p<0.01), and apnea (1/7 (14.2%) vs 2.2% (20/877), p=0.04)) were associated with higher mortality than children lacking these signs. We also observed anemia and malaria to have associations with mortality. Specifically, children who died, compared to survivors, had a 2.2 g/dL lower mean hemoglobin (7.8 g/dL [SD 3.0] vs 10.0 g/dL [SD 2.0], p<0.01). Children with a hemoglobin <5 mg/dL also had higher mortality than those with a 5 mg/dL or higher measurement (11.1% vs 2.1%, p<0.01). Similarly, children positive for malaria had a mortality of 5.4% while children testing negative had a lower 1.4% mortality rate (p<0.01). Children with malaria, compared to those testing negative, had a 1.9 mg/dL lower mean hemoglobin level (8.5 g/dL vs 10.4 g/dL, p<0.01).

Observational participants who died had a 1.7 cm lower mean MUAC than those who did not die (p<0.01). Notably, there was no difference between those who survived and

Confidential: For Review Only

those who did not based on the admission $SpO_2$ distribution (p=0.49), and although the point estimates for mortality were higher among children with a $SpO_2$ 90-92%, compared to 93-100%, these did not reach statistical significance (3.7% vs 2.2%, p=0.38).

## Discussion:

We identified potentially modifiable risk factors for mortality among an observational cohort of Malawian children with WHO-defined severe pneumonia and without severe malnutrition, HIV, or hypoxemia. We found those children who died were more likely to present with respiratory distress, anemia, malaria, and a lower MUAC. Our results also show that during the study period severe pneumonia was generally as frequent among Malawian children without high risk conditions as they are with them, and that about 20% of pneumonia deaths at Salima District Hospital during the study period may be attributed to lower risk children. Extrapolating these observations to broader estimates suggests >100,000 child pneumonia deaths annually in sub-Saharan Africa may occur among children without severe malnutrition, HIV, and hypoxemia.[10] Altogether, our results imply children with severe pneumonia lacking HIV, severe malnutrition, or hypoxemia are not only likely to be important in the broader child pneumonia epidemiological context but could benefit from more systematic scrutiny of four potentially modifiable domains of risk, (1) respiratory distress, (2) anemia, (3) malaria, and, (4) non-severe malnutrition, through more rigorous screening and testing.





Confidential: For Review Only

Currently, only MUAC is routinely recommended by the WHO IMCI guidelines, which are intended for use at frontline health facilities and hospital outpatient clinics that serve as the first point of contact for most patients.[5] Instead, guidelines recommend initially relying on caregiver report to identify difficult breathing, checking for anemia by examining the palms for pallor, and testing for malaria if fever is present (in endemic areas).[5] While severe malnutrition, HIV, and hypoxemia confer a higher mortality risk, there is value in identifying specific features that convey a higher probability of death among lower risk but severely ill patients. It is possible these children, if appropriately identified, may be more responsive to treatments and more likely to survive; the ability to target care would then be of benefit. For example, such children may be more successfully managed in hospital units with higher nurse to patient ratios that allow closer monitoring and more meticulous care. Results from CPAP IMPACT suggest that meaningful reductions in mortality among higher risk children with severe pneumonia may be challenging to widely achieve with available approaches in LMICs.

Previous studies from sub-Saharan Africa reported similar findings, though analyses aggregate children with and without high-risk conditions or severe and non-severe pneumonia. From Kenya a study of pediatric pneumonia hospitalizations sought to establish mortality rates and risk factors among children with pneumonia.[11] Among those with non-severe disease, pallor and a weight-for-age z-score <3 SD were associated with death, but having respirations 70 breaths/minute or higher, moderate malnutrition, moderate dehydration, or admission in a malaria-endemic region also increased mortality risk. Another Kenyan study of hospitalized children found overall



Confidential: For Review Only

mortality was 5%, and severe anemia, jaundice, chest indrawing, deep breathing, neurologic status, and axillary temperature (>39ºC or <36ºC) were predictive of immediate death.[12] In sum, these results were largely consistent with our findings and suggest that among lower-risk, acutely ill children the list of modifiable mortality risk factors may be limited. Severe respiratory distress, malaria, and severe anemia could be the most realistic targets.

In our observational cohort children who died were more likely to have a lower hemoglobin and were also more likely to have malaria than those who survived. As expected, a significant difference in mean hemoglobin existed between children with a positive versus negative malaria test. Given the syndromic approach to disease used by healthcare workers in LMICs, an overlap between primary respiratory illness and malaria has been shown in other studies. In a prospective cohort of children in Nigerian hospitals, 31% of those with pneumonia also had malaria,[13] while in Mozambique 19% of children with severe pneumonia had malaria.[14] In Uganda, a study of children attending 14 health centers found 30% had both pneumonia and malaria.[15] A large Kenyan cohort found 50% of children hospitalized with severe pneumonia had malaria at final diagnosis, while just 3% had both.[16]

Unfortunately, clinical assessment for pallor has been shown as largely unreliable for detecting anemia. A study of hospitalized Tanzanian children found an 8% sensitivity and 98% specificity for pallor identifying severe anemia.[17] Among children in Uganda and Bangladesh severe palmar and conjunctival pallor had sensitivities between 10-

Confidential: For Review Only

50% and a specificity of 99% for severe anemia.[18] Altogether, given the co-incidence of these conditions, the mortality risk associated with severe malaria and anemia, and the limited performance of pallor, routine hemoglobin and malaria testing among children with difficulty breathing seems warranted.

Unlike this study, prior inpatient and outpatient pediatric studies from Malawi found associations between moderately low $SpO_2$ ranges and increased odds of mortality among children with pneumonia.[19,20] The pediatric populations in these studies differed as they included children with severe malnutrition and/or HIV. Although in our study we also observed higher point estimates for mortality among children with a $SpO_2$ 90-92%, compared to 93-100%, this association did not reach statistical significance. Given these prior studies observed a $SpO_2$ and mortality relationship, it is possible our sample size was inadequate and a false negative type 2 error is present. It is also possible that severe malnutrition and/or HIV function as effect modifiers to this association. In a non-clinical trial context when HIV testing and anthropomorphic measurements are inconsistently performed effect modification may not be relevant.

There are two key limitations. First, the observational cohort experienced 21 deaths, and this limited our ability to fit multivariable models for mortality as planned. Second, although our prospective design and population of rural Malawian children at a district hospital should be considered strengths, this is a single center study. Increasing the certainty of our findings remains important and a larger, multi-center study is needed.



Confidential: For Review Only

In sum, we identified four risk factors for death (signs of respiratory distress, anemia, malaria, and lower MUAC) among Malawian children with severe pneumonia but without severe malnutrition, HIV infection or exposure, and hypoxemia. Based on these findings systematic screening of children with severe pneumonia for additional signs of respiratory distress, checking of hemoglobin levels, testing for malaria (when endemic), and measuring MUAC seem both feasible and warranted. Intervention studies examining the modifiability of these risk factors in similar populations are needed as is larger, confirmatory observational research.

Acknowledgements:

We thank the children and their caregivers who participated, Salima District Hospital and the Malawi Ministry of Health for their support of this research, and the entire CPAP IMPACT study team for their dedication and care of the children in this study. We also acknowledge the important contributions of Dr. Veena Billioux to this work.

Author contributions:

EDM acquired the funding. ME and EDM were responsible for conceptualization and design. EDM curated the data. DK and DM collected the data. DV, VB, ME, and EDM were responsible for data analysis. ME, EDM, TM, AGS, and MH were responsible for data interpretation. ME and EDM wrote the original draft. ME, EDM, TM, AGS, DK, DM, DV, and MH were responsible for writing, review, and editing.

Competing Interests Statement:

Confidential: For Review Only

We declare no competing interests.

Funding statement:

The Bill & Melinda Gates Foundation (OPP1123419), International AIDS Society (141022) and Health Empowering Humanity (award number not applicable) provided funding for this work.

Data availability statement:

Data are available upon reasonable request. A de-identified dataset and data dictionary will be provided after a signed data access agreement with the authors.

---

References:

1. Quantifying risks and interventions that have affected the burden of lower respiratory infections among children younger than 5 years: an analysis for the Global Burden of Disease Study 2017. *Lancet Infect Dis*. Jan 2020;20(1):60-79. doi:10.1016/s1473-3099(19)30410-4

2. Sonego M, Pellegrin MC, Becker G, Lazzerini M. Risk factors for mortality from acute lower respiratory infections (ALRI) in children under five years of age in low and middle-income countries: a systematic review and meta-analysis of observational studies. *PLoS One*. 2015;10(1):e0116380.

3. Lazzerini M, Sonego M, Pellegrin MC. Hypoxaemia as a Mortality Risk Factor in Acute Lower Respiratory Infections in Children in Low and Middle-Income Countries:



https://mc.manuscriptcentral.com/bmjpo

Confidential: For Review Only

Systematic Review and Meta-Analysis. *PLoS One*. 2015;10(9):e0136166. doi:10.1371/journal.pone.0116380

4. Theodoratou E, McAllister DA, Reed C, et al. Global, regional, and national estimates of pneumonia burden in HIV-infected children in 2010: a meta-analysis and modelling study. *Lancet Infect Dis*. Dec 2014;14(12):1250-8. doi:10.1016/s1473-3099(14)70990-9

5. WHO. Integrated Management of Childhood Illness: Chart Booklet. 2014;https://apps.who.int/iris/bitstream/handle/10665/104772/9789241506823_Chartbook_eng.pdf

6. McCollum ED, King C, Hammitt LL, et al. Reduction of childhood pneumonia mortality in the Sustainable Development era. *Lancet Respir Med*. Dec 2016;4(12):932-933. doi:10.1016/s2213-2600(16)30371-x

7. Smith AG, Eckerle M, Mvalo T, et al. CPAP IMPACT: a protocol for a randomised trial of bubble continuous positive airway pressure versus standard care for high-risk children with severe pneumonia using adaptive design methods. *BMJ Open Respir Res*. 2017;4(1):e000195. doi:10.1136/bmjresp-2017-000195

8. McCollum ED, Mvalo T, Eckerle M, et al. Bubble continuous positive airway pressure for children with high-risk conditions and severe pneumonia in Malawi: an open label, randomised, controlled trial. *Lancet Respir Med*. Nov 2019;7(11):964-974. doi:10.1016/s2213-2600(19)30243-7

9. WHO. Pocketbook of Hospital care for children. Guidelines for the management of common childhood illnesses. Second Edition.

Confidential: For Review Only

2013;https://apps.who.int/iris/bitstream/handle/10665/81170/9789241548373_eng.pdf?sequence=1

10. Liu L, Oza S, Hogan D, et al. Global, regional, and national causes of under-5 mortality in 2000-15: an updated systematic analysis with implications for the Sustainable Development Goals. *Lancet*. Dec 17 2016;388(10063):3027-3035. doi:10.1016/s0140-6736(16)31593-8

11. Agweyu A, Lilford RJ, English M. Appropriateness of clinical severity classification of new WHO childhood pneumonia guidance: a multi-hospital, retrospective, cohort study. *Lancet Glob Health*. Jan 2018;6(1):e74-e83. doi:10.1016/s2214-109x(17)30448-5

12. Berkley JA, Ross A, Mwangi I, et al. Prognostic indicators of early and late death in children admitted to district hospital in Kenya: cohort study. *Bmj*. Feb 15 2003;326(7385):361. doi:10.1136/bmj.326.7385.361

13. Graham H, Bakare AA, Ayede AI, et al. Diagnosis of pneumonia and malaria in Nigerian hospitals: A prospective cohort study. *Pediatr Pulmonol*. Jun 2020;55 Suppl 1(Suppl 1):S37-s50. doi:10.1002/ppul.24691

14. Sigaúque B, Roca A, Bassat Q, et al. Severe pneumonia in Mozambican young children: clinical and radiological characteristics and risk factors. *J Trop Pediatr*. Dec 2009;55(6):379-87. doi:10.1093/tropej/fmp030

15. Källander K, Nsungwa-Sabiiti J, Peterson S. Symptom overlap for malaria and pneumonia--policy implications for home management strategies. *Acta Trop*. Apr 2004;90(2):211-4. doi:10.1016/j.actatropica.2003.11.013

Confidential: For Review Only

16. Mwaniki MK, Nokes DJ, Ignas J, et al. Emergency triage assessment for hypoxaemia in neonates and young children in a Kenyan hospital: an observational study. *Bull World Health Organ*. Apr 2009;87(4):263-70. doi:10.2471/blt.07.049148

17. Mogensen CB, Sørensen JE, Bjorkman A. Pallor as a sign of anaemia in small Tanzanian children at different health care levels. *Acta Trop*. Oct 2006;99(2-3):113-8. doi:10.1016/j.actatropica.2005.12.010

18. Kalter HD, Burnham G, Kolstad PR, et al. Evaluation of clinical signs to diagnose anaemia in Uganda and Bangladesh, in areas with and without malaria. *Bull World Health Organ*. 1997;75 Suppl 1(Suppl 1):103-11.

19. Colbourn T, King C, Beard J, et al. Predictive value of pulse oximetry for mortality in infants and children presenting to primary care with clinical pneumonia in rural Malawi: A data linkage study. *PLoS Med*. Oct 2020;17(10):e1003300. doi:10.1371/journal.pmed.1003300

20. Hooli S, Colbourn T, Lufesi N, et al. Predicting Hospitalised Paediatric Pneumonia Mortality Risk: An External Validation of RISC and mRISC, and Local Tool Development (RISC-Malawi) from Malawi. *PLoS One*. 2016;11(12):e0168126. doi:10.1371/journal.pone.0168126

Confidential: For Review Only

Table 1. Characteristics of children 1-59 months old with WHO-defined severe pneumonia at Salima District Hospital, Malawi in an observational study compared to a randomized controlled trial CPAP IMPACT

| Characteristic | Observational study N=884 | CPAP IMPACT N=644 | p-value |
|---|---|---|---|
| Age in months, median (IQR) | 9.2 (4.7, 17.2) | 7.7 (3.1, 15.2) | <0.01 |
| Females, n (%) | 362 (41.0%) | 299 (46.4%) | 0.03 |
| Males, n (%) | 522 (59.0%) | 345 (53.6%) | |
| Weight in kg, mean (SD) | 8.5 (2.5) | 7.2 (2.8) | <0.01 |
| MUAC in cm, mean (SD) | 14.0 (1.2) | 12.7 (1.9) | <0.01 |
| HIV-infected, n (%) | 0 (0.0%) | 36 (5.6%) | <0.01 |
| HIV-exposed uninfected, n (%) | 0 (0.0%) | 99 (15.4%) | <0.01 |
| Severe malnutrition, n (%) | 0 (0.0%) | 222 (34.5%) | <0.01 |
| Hypoxemia only, n (%) | 0 (0.0%) | 287 (44.6%) | <0.01 |
| Pulse rate in beats/min, mean (SD) | 162.4 (20.7) | 164.1 (24.0) | 0.15 |
| Respiratory rate in breaths/min, mean (SD) | 59.6 (12.9) | 61.9 (15.1) | <0.01 |
| $SpO_2$, median (IQR) | 96 (95, 98) | 88 (83, 94) | <0.01 |
| $SpO_2$ 93-100%, n (%) | 805 (91.1%) | 191 (29.7%) | |
| $SpO_2$ 90-92%, n (%) | 79 (8.9%) | 38 (5.9%) | |
| $SpO_2$ <90%, n (%) | 0 (0.0%) | 415 (64.4%) | |
| 1 or more respiratory danger signs[1], n (%) | 866 (98.0%) | 638 (99.1%) | 0.09 |
| Severe chest indrawing, n (%) | 777 (87.9%) | 575 (89.3%) | 0.41 |
| Head nodding, n (%) | 188 (21.3%) | 176 (27.3%) | <0.01 |
| Grunting, n (%) | 112 (12.7%) | 154 (23.9%) | <0.01 |
| Very fast breathing for age, n (%)[2] | 256 (29.0%) | 211 (32.8%) | 0.11 |
| Stridor when calm, n (%) | 14 (1.6%) | 13 (2.0%) | 0.52 |
| Apnea, n (%) | 7 (0.8%) | 37 (5.7%) | <0.01 |
| Nasal flaring, n (%) | 519 (58.7%) | 471 (73.1%) | <0.01 |
| Convulsions, n (%) | 45 (5.1%) | 61 (9.5%) | <0.01 |
| Inability to feed, n (%) | 27 (3.1%) | 78 (12.1%) | <0.01 |
| Vomiting everything, n (%) | 12 (1.4%) | 19 (3.0%) | 0.03 |
| Blantyre Coma Score ≤4, n (%) | 20 (2.3%) | 55 (8.5%) | <0.01 |
| Crackles on auscultation, n (%) | 429 (48.5%) | 311 (48.3%) | 0.95 |
| Wheeze on auscultation, n (%) | 160 (18.1%) | 128 (19.9%) | 0.38 |
| Hb <5 g/dL, n (%) | 27 (3.1%) | 50 (7.8%) | <0.01 |
| Hb (g/dL), mean (SD) | 10.0 (2.1) | 9.5 (2.6) | <0.01 |
| Malaria positive, n (%)[3] | 204 (23.3%) | 197 (30.6%) | <0.01 |
| Death, n (%) | 21 (2.4%) | 88 (13.7%) | <0.01 |

Confidential: For Review Only

WHO indicates World Health Organization; IQR, interquartile range; SD, standard deviation; MUAC, mid-upper arm circumference; $SpO_2$; peripheral oxyhemoglobin saturation; Hb, hemoglobin.

[1]Any of the following signs of respiratory distress: severe chest indrawing, head nodding, grunting, very fast breathing for age, stridor when calm, apnea, nasal flaring

[2]$\geq$80 breaths/min if 30-59 days old, $\geq$70 breaths/min if 2-11 months old, and $\geq$60 breaths/min if 12-59 months old

[3]Rapid test positive, 10 observational study participants were missing test results

Confidential: For Review Only

Table 2. Association between hospitalization characteristics and outcome children 1-59 months old with WHO-defined severe pneumonia without HIV, severe malnutrition, and/or hypoxemia in an observational study

| Characteristic | | Survived N=863 | Died N=21 | p-value |
|---|---|---|---|---|
| Age in months, median (IQR) | | 9.2 (4.7, 17.3) | 8.1 (6.5, 12.0) | 0.74 |
| Sex, n (%) | Females | 353 (97.5%) | 9 (2.5%) | 0.86 |
| | Males | 510 (97.7%) | 12 (2.3%) | |
| Respiratory rate (breaths/min), mean (SD) | | 59.6 (12.9) | 60.3 (13.7) | 0.82 |
| Fast breathing for age, n (%) | Yes | 720 (97.6%) | 18 (2.4%) | 0.78 |
| | No | 143 (97.9%) | 3 (2.1%) | |
| Pulse rate (beats/min), mean (SD) | | 162.2 (20.4) | 170.0 (30.4) | 0.09 |
| Pulse rate >180 beats/min, n (%) | Yes | 113 (95.0%) | 6 (5.0%) | 0.14 |
| | No | 400 (97.6%) | 10 (2.4%) | |
| Pulse rate >160 beats/min, n (%) | Yes | 165 (97.6%) | 4 (2.4%) | 0.15 |
| | No | 184 (99.5%) | 1 (0.5%) | |
| Severe chest indrawing, n (%) | Yes | 760 (97.8%) | 17 (2.2%) | 0.32 |
| | No | 103 (96.3%) | 4 (3.7%) | |
| Head nodding, n (%) | Yes | 180 (95.7%) | 8 (4.3%) | 0.06 |
| | No | 683 (98.1%) | 13 (1.9%) | |
| Grunting, n (%) | Yes | 102 (91.1%) | 10 (8.9%) | <0.01 |
| | No | 760 (98.6%) | 11 (1.4%) | |
| Stridor when calm, n (%) | Yes | 12 (85.7%) | 2 (14.3%) | <0.01 |
| | No | 851 (97.8%) | 19 (2.2%) | |
| Apnea, n (%) | Yes | 6 (85.7%) | 1 (14.3%) | 0.04 |
| | No | 857 (97.7%) | 20 (2.3%) | |
| Nasal flaring, n (%) | Yes | 507 (97.6%) | 12 (2.3%) | 0.88 |
| | No | 356 (97.5%) | 9 (2.5%) | |
| MUAC in cm, mean (SD) | | 14.0 (1.2) | 13.3 (1.0) | <0.01 |
| MUAC 11.5-13.5 cm, n (%) | Yes | 255 (96.2%) | 10 (3.8%) | 0.07 |
| | No | 608 (98.2%) | 11 (1.8%) | |
| Hb (g/dL), mean (SD) | | 10.0 (2.0) | 7.8 (3.0) | <0.01 |
| Hb <5 g/dL | Yes | 24 (88.9%) | 3 (11.1%) | <0.01 |
| | No | 839 (99.6%) | 18 (2.%) | |
| Malaria positive, n (%)[1] | Yes | 193 (94.6%) | 11 (5.4%) | <0.01 |
| | No | 663 (98.5%) | 10 (1.5%) | |
| $SpO_2$ (%), median (IQR) | | 96 (95, 98) | 96 (94, 98) | 0.49 |
| $SpO_2$ | 93-100% | 787 (97.8%) | 18 (2.2%) | 0.38 |
| | 90-92% | 76 (96.2%) | 3 (3.7%) | |

Confidential: For Review Only

WHO indicates World Health Organization; IQR, interquartile range; SD, standard deviation; MUAC, mid-upper arm circumference; Hb, hemoglobin.

[1]10 participants were missing test results

Confidential: For Review Only

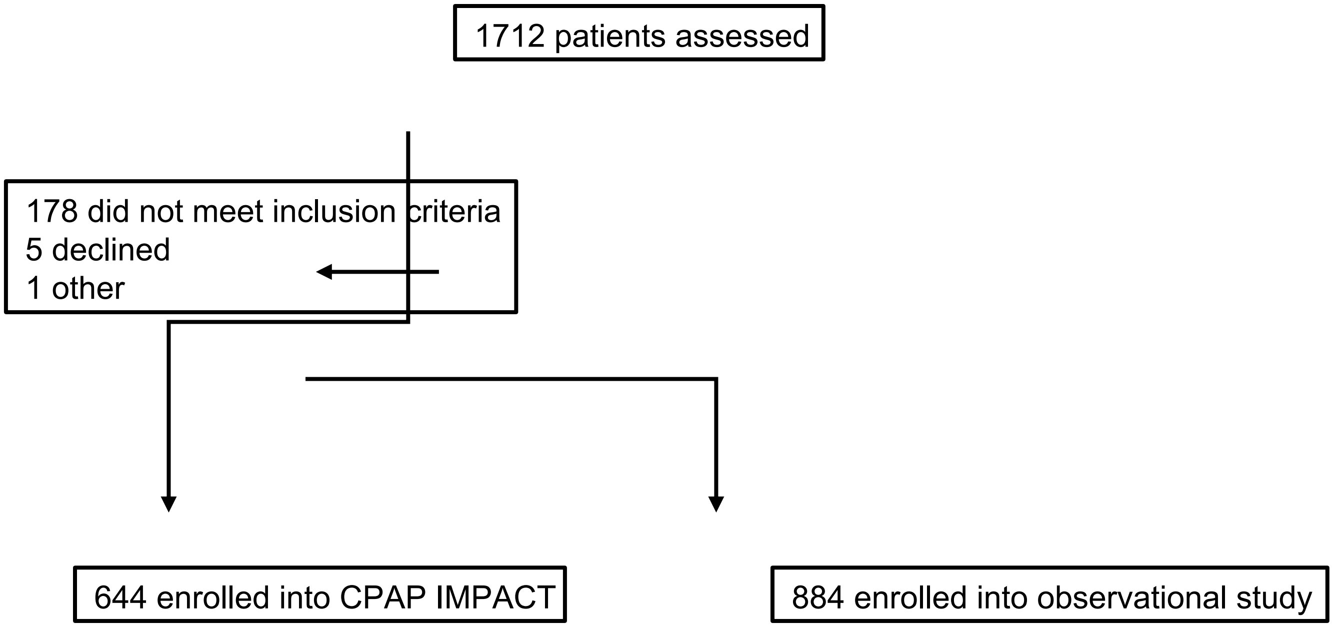
1712 patients assessed
178 did not meet inclusion criteria
5 declined
1 other
644 enrolled into CPAP IMPACT
884 enrolled into observational study

BMJ Paediatrics Open

# Identifying modifiable risk factors for mortality in children aged 1-59 months admitted with WHO-defined severe pneumonia: a single centre observational cohort study from rural Malawi



| | |
|---|---|
| Journal: | *BMJ Paediatrics Open* |
| Manuscript ID | bmjpo-2021-001330.R1 |
| Article Type: | Original research |
| Date Submitted by the Author: | 05-Jan-2022 |





| | |
|---|---|
| Complete List of Authors: | Eckerle, Michelle; Cincinnati Children's Hospital Medical Center, Pediatrics<br>Mvalo, Tisungane; University of North Carolina Project; University of North Carolina at Chapel Hill School of Medicine, Department of Pediatrics<br>Smith, Andrew; The University of Utah School of Medicine<br>Kondowe, Davie; University of North Carolina Project<br>Makonokaya, Don; University of North Carolina Project<br>Vaidya, Dhananjay; Johns Hopkins University School of Medicine<br>Hosseinipour, Mina; University of North Carolina Project; University of North Carolina at Chapel Hill School of Medicine<br>McCollum, Eric D.; Johns Hopkins School of Medicine, Department of Paediatrics |





| | |
|---|---|
| Keywords: | Epidemiology, Health services research, Mortality |
| | |



SCHOLARONE™
Manuscripts

BMJ

*I, the Submitting Author has the right to grant and does grant on behalf of all authors of the Work (as defined in the below author licence), an exclusive licence and/or a non-exclusive licence for contributions from authors who are: i) UK Crown employees; ii) where BMJ has agreed a CC-BY licence shall apply, and/or iii) in accordance with the terms applicable for US Federal Government officers or employees acting as part of their official duties; on a worldwide, perpetual, irrevocable, royalty-free basis to BMJ Publishing Group Ltd ("BMJ") its licensees and where the relevant Journal is co-owned by BMJ to the co-owners of the Journal, to publish the Work in this journal and any other BMJ products and to exploit all rights, as set out in our licence.*

*The Submitting Author accepts and understands that any supply made under these terms is made by BMJ to the Submitting Author unless you are acting as an employee on behalf of your employer or a postgraduate student of an affiliated institution which is paying any applicable article publishing charge ("APC") for Open Access articles. Where the Submitting Author wishes to make the Work available on an Open Access basis (and intends to pay the relevant APC), the terms of reuse of such Open Access shall be governed by a Creative Commons licence – details of these licences and which Creative Commons licence will apply to this Work are set out in our licence referred to above.*

*Other than as permitted in any relevant BMJ Author's Self Archiving Policies, I confirm this Work has not been accepted for publication elsewhere, is not being considered for publication elsewhere and does not duplicate material already published. I confirm all authors consent to publication of this Work and authorise the granting of this licence.*

Confidential: For Review Only

Identifying modifiable risk factors for mortality in children aged 1-59 months admitted with WHO-defined severe pneumonia: a single centre observational cohort study from rural Malawi

Michelle Eckerle, MD[1,2]

Tisungane Mvalo, FC Paed[3,4]

Andrew G. Smith, MD[5]

Davie Kondowe, BBiomedSc[3]

Don Makonokaya, BScPH[3]

Dhananjay Vaidya, PhD[6]

Mina C. Hosseinipour, MD[3,8]

Eric D. McCollum, MD, MPH[9,10]

[1]Division of Emergency Medicine, Cincinnati Children's Hospital Medical Center, Cincinnati, Ohio, USA

[2]Department of Pediatrics, University of Cincinnati College of Medicine, Cincinnati, Ohio, USA

[3]University of North Carolina Project Malawi, Lilongwe, Malawi

[4]Department of Pediatrics, School of Medicine, University of North Carolina at Chapel Hill, NC, USA.

[5]Department of Pediatrics, University of Utah School of Medicine, Salt Lake City, Utah, USA

Confidential: For Review Only

[6]Department of Medicine, Epidemiology and the BEAD Core, Johns Hopkins University, Baltimore, MD, USA

[7]Department of Pediatrics, BEAD Core, Johns Hopkins University, Baltimore, MD, USA

[8]Division of Infectious Disease, University of North Carolina at Chapel Hill, Chapel Hill, North Carolina, USA

[9]Global Program for Pediatric Respiratory Sciences, Eudowood Division of Pediatric Respiratory Sciences, Department of Pediatrics, School of Medicine, Johns Hopkins University, Baltimore, Maryland, United States

[10]Department of International Health, Bloomberg School of Public Health, Johns Hopkins University, Baltimore, Maryland, United States

Corresponding author:

Eric D. McCollum, MD, MPH

Email: emccoll3@jhmi.edu

Word count: 2,494

Confidential: For Review Only

Abstract:

Objective: Although HIV-infection, severe malnutrition, and hypoxemia are associated with high mortality in children with World Health Organization (WHO)-defined severe pneumonia in sub-Saharan Africa, many do not have these conditions and yet mortality remains elevated compared to high-resource settings. Further stratifying mortality risk for children without these conditions could permit more strategic resource utilization and improved outcomes. We therefore evaluated associations between mortality and clinical characteristics not currently recognized by the WHO as high-risk among children in Malawi with severe pneumonia but without HIV (including exposure), severe malnutrition, and hypoxemia.

Methods: Between May 2016 and March 2018 we conducted a prospective observational study alongside a randomized controlled trial (CPAP IMPACT) at Salima District Hospital in Malawi. Children 1-59 months hospitalized with WHO-defined severe pneumonia without severe malnutrition, HIV, and hypoxemia were enrolled. Study staff assessed children at admission and ascertained hospital outcomes. We compared group characteristics using Student t, rank sum, Chi-Square or Fisher's Exact tests as appropriate.

Results: Among 884 participants grunting (10/112 (8.9%) vs 11/772 (1.4%)), stridor (2/14 (14.2%) vs 19/870 (2.1%)), apnea (1/7 (14.2%) vs 20/877 (2.2%)), hemoglobin <5 g/dL (3/27 (11.1%) vs 18/857 (2.1%)), and malaria (11/204 (5.3%) vs 10/680 (1.4%)) were associated with mortality compared to children without these characteristics.



Confidential: For Review Only

Children who survived had a 2.2 g/dL higher mean hemoglobin and 0.7 cm higher mean mid-upper arm circumference (MUAC) than those who died.

Conclusion: In this single-center study, our analysis identifies potentially modifiable risk factors for mortality among hospitalized Malawian children with severe pneumonia: specific signs of respiratory distress (grunting, stridor, apnea), hemoglobin <5 g/dL, and malaria infection. Significant differences in mean hemoglobin and MUAC were observed between those who survived and those who died. These factors could further stratify mortality risk among hospitalized Malawian children with severe pneumonia lacking recognized high-risk conditions.

Confidential: For Review Only

What is known about this subject

- Lower respiratory infections like pneumonia kill an estimated 800,000 children annually, making it the number one infectious cause of pediatric deaths globally.
- Among children with pneumonia, important drivers of mortality in low-income and middle-income countries (LMICs) are severe malnutrition, HIV infection or exposure, and hypoxemia.
- Children in LMICs with severe pneumonia but without HIV (including exposure), severe malnutrition, and hypoxemia may account for a large proportion of the overall pneumonia burden and mortality but data is limited.

What this study adds

- About 20% of pneumonia deaths at one rural district hospital in Malawi over two years were attributable to children lacking HIV, severe malnutrition, and hypoxemia.
- Among Malawian children with severe pneumonia without HIV, severe malnutrition, and hypoxemia, respiratory distress (apnea, stridor, grunting), hemoglobin <5 g/dL, and malaria predicted mortality.
- Significant differences in mean hemoglobin and mid-upper arm circumference were present in severe pneumonia cases lacking recognized high-risk conditions who survived or died.

Confidential: For Review Only

## Introduction

Pneumonia causes approximately 800,000 deaths outside of the neonatal period among under 5 year olds worldwide and this burden is disproportionately borne in low-income and middle-income countries (LMICs).[1] Severe malnutrition, HIV-infection or exposure, and hypoxemia (peripheral oxyhemoglobin saturation ($SpO_2$) <90%) place children with pneumonia at higher risk of death.[1-4] While children with severe pneumonia but without these conditions may have relatively lower mortality, they still may comprise a substantial overall burden of pneumonia morbidity and mortality in LMICs.[1] For example, in Malawi approximately half of child pneumonia deaths were attributable to HIV and almost 90% of hospitalized pneumonia were among children without hypoxemia.[4,5] In a multi-country severe pneumonia study 61% of deaths occurred among children without severe malnutrition.[6] Identifying clinical characteristics that predict mortality, can be objectively measured, and may be modifiable among pediatric pneumonia cases lacking severe malnutrition, HIV (including exposure), and hypoxemia could represent an effective strategy for lowering pneumonia deaths in LMICs.

The World Health Organization (WHO) recommends a syndromic approach to child pneumonia diagnosis and management in LMICs to improve implementation feasibility.[7] Recommended treatment for WHO-defined severe pneumonia includes hospitalization, parenteral antibiotics, and oxygen.[7] Understanding which children with WHO-defined severe pneumonia in LMICs are at high risk of death is important as it can impact resource allocation in austere settings where advanced care and other resources are limited.[8]



Confidential: For Review Only

In conjunction with a randomized controlled trial called CPAP IMPACT, which studied children with WHO-defined severe pneumonia and at least one high-risk condition (severe malnutrition, HIV-infection or exposure, and/or hypoxemia),[9,10] we conducted a parallel prospective observational study of children with WHO-defined severe pneumonia but without any of these three high-risk conditions. Our main objectives were to contextualize observational study participants through their clinical characteristics and outcomes, and then to identify clinical predictors of mortality with a priority towards factors that may be objectively measured, potentially modifiable, and not included in WHO pneumonia guidelines.

## Methods

We conducted a pre-specified sub-study in conjunction with a randomized controlled trial (CPAP IMPACT).[9,10] CPAP IMPACT compared bubble continuous positive airway pressure (bCPAP) to low flow oxygen for children with WHO-defined severe pneumonia and at least one high-risk condition. From May 2016 through March 2018, children with WHO-defined severe pneumonia and ineligible for CPAP IMPACT were invited to participate in this ancillary prospective observational study. The study took place at Salima District Hospital in Salima, Malawi, a secondary hospital lacking intensive care resources. Institutional review boards approved the protocol. Enrolled participants provided written consent.





Confidential: For Review Only

During the study period children admitted to Salima District Hospital were screened for eligibility at all times. Children were eligible if 1-59 months with WHO-defined severe pneumonia and without any of three high-risk conditions: (1) HIV-infection (<12 months old with a positive HIV DNA PCR or ≥12 months old with HIV antibodies) or exposure (<24 months old born to a HIV-infected mother and not meeting HIV-infection criteria), (2) severe malnutrition (weight for age z score < -3 standard deviations (SD), mid-upper arm circumference (MUAC) <11.5 cm, and/or bilateral pedal edema), or (3) a $SpO_2$ <90%.

Per WHO guidelines, children could meet severe pneumonia criteria by having cough and/or difficult breathing plus signs of pneumonia with at least one general danger sign or signs of respiratory distress with or without a general danger sign.[11] Respiratory distress included head nodding, nasal flaring, grunting, stridor when calm, tracheal tugging, severe chest indrawing, very fast breathing for age (≥80 breaths/min if 30-59 days old, ≥70 breaths/min if 2-11 months old, and ≥60 breaths/min if 12-59 months old), or apnea. Signs of pneumonia included fast breathing (60-79 breaths/min if 30-59 days old, 50-69 breaths/min if 2-11 months old, and 40-59 breaths/min if 12-59 months old) and/or chest indrawing. Inability to feed, vomiting everything, lethargic or unconscious (Blantyre Coma Score ≤4),[12] and convulsions comprised general danger signs.

Consistent with national guidelines malaria infection was confirmed on all participants at enrollment using rapid antigen testing (SD Bioline (HRP2/pLDH, sensitivity 97.4%-99.7%, specificity 99.3%-99.7%)) as routine microscopy was limited.[13] Anemia was also

Confidential: For Review Only

assessed by point-of-care testing (Hemocue 301+). A Rad5 pulse oximeter (Masimo, Irvine, CA, USA) measured the $SpO_2$ on children breathing in room air. Study staff recommended initial evaluations and treatments at enrollment and thereafter care was led by government hospital staff, including oxygen, antibiotics, and fluids. According to local guidelines, all patients received benzylpenicillin and gentamicin and could be switched to ceftriaxone for treatment failure.[14] Similarly, per local guidelines, severe malaria was treated with artesunate and uncomplicated malaria by lumefantrine-artemether.[13] Children were followed passively by study staff until hospital outcome. This study's primary outcome was in-hospital mortality.

*Statistical Analysis*

Bivariable analysis comparing clinical variables with in-hospital mortality was performed as follows: The Student's *t*-test was used to assess differences between the means of normally distributed continuous variables (tabulated as means and standard deviations (SDs)), the non-parametric Mann-Whitney U test evaluated differences in the rank sums of continuous variables (tabulated as medians and interquartile range (IQR) presented as 25$^{th}$ and 75$^{th}$ percentile, Chi Square and Fisher's Exact tests (if the large sample assumption could not be justified) analyzed categorical clinical variables (tabulated as numbers and percentages). To understand whether an association existed between mortality and the number of significant risk factors per patient, logistic regression was performed. Although we planned to fit multivariable regression models for mortality we determined it was not advisable due to the number of mortality events experienced. All analyses were conducted using Stata (version 15.1; StataCorp, College Station, Texas).

Confidential: For Review Only

## Patient and public involvement

Patients and the public in Malawi were sensitized to the development, design, recruitment and conduct via local meetings before, during, and after the research.

## Results

From May 2016 enrollment began for the observational study and concluded at the same time as the parent CPAP IMPACT trial in March 2018 (Figure 1). Among 1,062 children that did not meet inclusion criteria for CPAP IMPACT, 884 met criteria for the observational study and agreed to participate.

We sought to identify whether any participant characteristics – with an emphasis on objective and potentially modifiable factors not included in the WHO pneumonia guidelines[7] – had associations with hospital mortality (Table 1). Children with respiratory distress signs like grunting (10/112 (8.9%) vs 11/772 (1.4%), p<0.01), stridor when calm (2/14 (14.2%) vs 19/870 (2.1%), p<0.01), and apnea (1/7 (14.2%) vs 2.2% (20/877), p=0.04)) had higher mortality rates than children without these signs. Anemia and malaria were also associated with mortality. Specifically, children who died, compared to survivors, had a 2.2 g/dL lower mean hemoglobin (7.8 g/dL [SD 3.0] vs 10.0 g/dL [SD 2.0], p<0.01). Children with a hemoglobin <5 mg/dL also had higher mortality than those with a 5 mg/dL or higher measurement (11.1% vs 2.1%, p<0.01). Children positive for malaria had a mortality of 5.4% while children testing negative had a mortality rate of 1.4% (p<0.01). Children with malaria, compared to those testing negative, had a 1.9



Confidential: For Review Only

mg/dL lower mean hemoglobin level (8.5 g/dL vs 10.4 g/dL, p<0.01). In a sensitivity analysis excluding seven children without a malaria test result, the overall findings were consistent except for the relationship between apnea and death. This association was qualitatively similar but did not reach statistical significance (p=0.16, Supplemental Table 1).

Participants who died had a 1.7 cm lower mean MUAC than those who did not die (p<0.01). Notably, there was no difference between those who survived and those who did not based on the admission $SpO_2$ distribution (p=0.49), and although the point estimates for mortality were higher among children with a $SpO_2$ 90-92%, compared to 93-100%, these did not reach statistical significance (3.7% vs 2.2%, p=0.38).

Of the identified risk factors for mortality, we considered dichotomous factors (apnea, stridor when calm, grunting, malaria status, hemoglobin <5 g/dL) and used logistic regression to determine whether the odds of mortality increased with increasing numbers of risk factors per patient. The maximum number of risk factors observed for any child was four. Compared to children with only one risk factor, children with two, three, or four risk factors had an increased odds ratio for death that increased with an increasing number of risk factors (Table 2).

In Supplemental Table 2 we compared observational participants to children enrolled in CPAP IMPACT to provide context for both groups as well as the spectrum of pneumonia at Salima District Hospital during the study period. The mortality of





Confidential: For Review Only

observational children was 11.3 absolute percentage points lower than those in CPAP IMPACT (2.4% vs 13.7%, p<0.01). Altogether, when accounting for trial participants and the observational group, the hospital mortality of severe pneumonia was 7.1% (109/1,528).

## Discussion:

We identified potentially modifiable, objectively measurable risk factors for mortality among an observational cohort of Malawian children with WHO-defined severe pneumonia and without severe malnutrition, HIV, or hypoxemia. We found those children who died were more likely to present with respiratory distress, anemia, malaria, and a lower MUAC. Our results also show that during the study period severe pneumonia was generally as frequent among Malawian children without high risk conditions as they were with them, and that about 20% of pneumonia deaths at Salima District Hospital during this period were attributable to lower risk children. Assuming these data are representative then potentially high numbers of pneumonia deaths among sub-Saharan African children without severe malnutrition, HIV, and hypoxemia may occur each year.[15] Altogether, if confirmed, our results imply such children could be important in the broader pneumonia epidemiological context and may benefit from more systematic scrutiny of these additional, potentially modifiable domains of risk, (1) respiratory distress, (2) anemia, (3) malaria, and, (4) non-severe malnutrition, through more rigorous screening and testing.

Confidential: For Review Only

Currently, only MUAC is routinely recommended by the WHO IMCI guidelines for use at frontline health facilities and hospital outpatient clinics serving as an entry point for most patients.[7] Instead, guidelines recommend using caregiver report to identify difficult breathing, checking for anemia by examining for pallor, and testing for malaria if fever is present (in endemic areas).[7] While severe malnutrition, HIV, and hypoxemia confer a higher mortality risk, there is value in identifying specific features that convey a higher probability of death among lower risk but severely ill patients. It is possible these children, if appropriately identified, may be more treatment responsive and more likely to survive; the ability to target care would be beneficial. For example, such children may be more successfully managed if cohorted into hospital units with higher nurse to patient ratios that allow closer monitoring and more meticulous care. Results from CPAP IMPACT suggest meaningful reductions in mortality among higher risk children with severe pneumonia may be challenging to achieve with available approaches in LMICs.

Previous studies reported similar findings to this study, though analyses aggregate children with and without high-risk conditions or severe and non-severe pneumonia. A large meta-analysis of children from LMICs found that young age (<2 months), *Pneumocystis jirovecii* infection, chronic underlying diseases, HIV/AIDS and severe malnutrition all increased the likelihood of death.[2] Within sub-Saharan Africa, a Kenyan study of pediatric pneumonia hospitalizations sought to establish mortality rates and risk factors among children with pneumonia.[16] Among those with non-severe disease, pallor and a weight-for-age z-score <3 SD were associated with death, but having respirations

Confidential: For Review Only

≥70 breaths/minute, moderate malnutrition, moderate dehydration, or admission in a malaria-endemic region also increased mortality risk. Another Kenyan study of hospitalized children found overall mortality was 5%, and severe anemia, jaundice, chest indrawing, deep breathing, neurologic status, and axillary temperature (>39ºC or <36ºC) were predictive of immediate death.[17] Of note, a recent study used a large pooled dataset to test three different risk scores for pneumonia mortality prediction among hospitalized children; the authors concluded that these risk scores inadequately performed when externally validated.[18]

In our observational cohort children who died were more likely to have a lower hemoglobin and were also more likely to have malaria than those who survived. As expected, a significant difference in mean hemoglobin existed between children with a positive versus negative malaria test. Given the syndromic approach to disease used by healthcare workers in LMICs, an overlap between primary respiratory illness and malaria has been previously reported. In a prospective cohort of children in Nigerian hospitals, 31% of those with pneumonia also had malaria,[19] while in Mozambique 19% of children with severe pneumonia had malaria.[20] In Uganda, a study of children attending 14 health centers found 30% had both pneumonia and malaria.[21] A large Kenyan cohort of hospitalized hypoxemic children found a final diagnosis of malaria more frequently than lower respiratory tract infection (35% versus 32%).[22]

Unfortunately, clinical pallor has been shown as unreliable for detecting anemia. A study of hospitalized Tanzanian children found 8% sensitivity and 98% specificity for pallor



Confidential: For Review Only

identifying severe anemia.[23] Among children in Uganda and Bangladesh severe palmar and conjunctival pallor had sensitivities between 10%-50% and a specificity of 99% for severe anemia.[24] Altogether, given the co-incidence of these conditions, the mortality risk associated with severe malaria and anemia, and the limited performance of pallor, routine hemoglobin and malaria testing among children with difficulty breathing merits further consideration.

Unlike this study, prior inpatient pediatric studies from Malawi found associations between moderately low $SpO_2$ ranges and increased odds of mortality among children with pneumonia (between 50% and 330% higher).[25,26] The pediatric populations in these studies differed as they included severe malnutrition and/or HIV cases. Although in our study we also observed higher point estimates for mortality among children with a $SpO_2$ 90-92%, compared to 93-100%, this association did not reach statistical significance. We expect that with a larger sample size a similar association between $SpO_2$ and mortality would have been observed in this study.

There are several limitations. First, malaria detection was determined by rapid antigen testing rather than peripheral blood microscopy. While children in endemic settings may have a positive rapid test due to persistent antigenemia rather than an acute infection,[27] our use of rapid testing was consistent with locally recommended practice in Malawi, a setting with limited microscopy capacity.[13] Second, the cohort experienced 21 deaths, and this restricted our ability to fit multivariable models for mortality as planned. Third, although our prospective design and population of rural Malawian children at a district



Confidential: For Review Only

hospital should be considered strengths, this is a single center study. Increasing the certainty of our findings remains important and a larger, multi-center study is needed.

In sum, we identified multiple risk factors for death (specific signs of respiratory distress (stridor, apnea, grunting), hemoglobin <5 g/dL, and malaria) among Malawian children with severe pneumonia but without severe malnutrition, HIV infection or exposure, and hypoxemia. Children who died had a lower MUAC than those who survived, but statistically significant thresholds were not apparent. Based on these findings systematic screening of children with severe pneumonia for additional signs of respiratory distress, checking of hemoglobin levels, testing for malaria (when endemic), and measuring MUAC, followed by closer monitoring and standard treatment for any identified abnormalities, seem feasible and warrant further consideration. Intervention studies examining the modifiability of these risk factors in similar populations are needed as is larger, confirmatory observational research in other LMIC settings.

Acknowledgements:

We thank the children and their caregivers who participated, Salima District Hospital and the Malawi Ministry of Health for their support of this research, and the entire CPAP IMPACT study team for their dedication and care of the children in this study. We also acknowledge the important contributions of Dr. Veena Billioux to this work.

Author contributions:

Confidential: For Review Only

EDM acquired the funding. ME and EDM were responsible for conceptualization and design. EDM curated the data. DK and DM collected the data. DV, VB, ME, and EDM were responsible for data analysis. ME, EDM, TM, AGS, and MH were responsible for data interpretation. ME and EDM wrote the original draft. ME, EDM, TM, AGS, DK, DM, DV, and MH were responsible for writing, review, and editing. EDM is the guarantor.

Competing Interests Statement:

We declare no competing interests.

Funding statement:

The Bill & Melinda Gates Foundation (OPP1123419), International AIDS Society (141022) and Health Empowering Humanity (award number not applicable) provided funding for this work.

Data availability statement:

Data are available upon reasonable request. A de-identified dataset and data dictionary will be provided after a signed data access agreement with the authors.

References:

1. GBD 2017 Lower Respiratory Infections Collaborators. Quantifying risks and interventions that have affected the burden of lower respiratory infections among children younger than 5 years: an analysis for the Global Burden of Disease Study 2017. *Lancet Infect Dis* 2020;**20**:60-79.

Confidential: For Review Only

2. Sonego M, Pellegrin MC, Becker G, Lazzerini M. Risk factors for mortality from acute lower respiratory infections (ALRI) in children under five years of age in low and middle-income countries: a systematic review and meta-analysis of observational studies. *PLoS One* 2015;**10**:e0116380.

3. Lazzerini M, Sonego M, Pellegrin MC. Hypoxaemia as a Mortality Risk Factor in Acute Lower Respiratory Infections in Children in Low and Middle-Income Countries: Systematic Review and Meta-Analysis. *PLoS One* 2015;**10**:e0136166.

4. Theodoratou E, McAllister DA, Reed C, et al. Global, regional, and national estimates of pneumonia burden in HIV-infected children in 2010: a meta-analysis and modelling study. *Lancet Infect Dis* 2014;**14**:1250-8.

5. McCollum ED, Nambiar B, Deula R, et al. Impact of the 13-Valent Pneumococcal Conjugate Vaccine on Clinical and Hypoxemic Childhood Pneumonia over Three Years in Central Malawi: An Observational Study. *PLoS One* 2017;**12**:e0168209.

6. Gallagher KE, Knoll MD, Prosperi C, et al. The predictive performance of a pneumonia severity score in HIV-negative children presenting to hospital in seven low and middle-income countries. *Clin Infect Dis* 2020;**70**:1050–1057.

7. WHO. Integrated Management of Childhood Illness: Chart Booklet. 2014. Available: https://apps.who.int/iris/bitstream/handle/10665/104772/9789241506823_Chartbook_eng.pdf [Accessed 28 Dec 2021]

8. McCollum ED, King C, Hammitt LL, et al. Reduction of childhood pneumonia mortality in the Sustainable Development era. *Lancet Respir Med* 2016;**4**:932-933.

Confidential: For Review Only

9. Smith AG, Eckerle M, Mvalo T, et al. CPAP IMPACT: a protocol for a randomised trial of bubble continuous positive airway pressure versus standard care for high-risk children with severe pneumonia using adaptive design methods. *BMJ Open Respir Res* 2017;**4**:e000195.

10. McCollum ED, Mvalo T, Eckerle M, et al. Bubble continuous positive airway pressure for children with high-risk conditions and severe pneumonia in Malawi: an open label, randomised, controlled trial. *Lancet Respir Med* 2019;**7**:964-974.

11. WHO. Pocketbook of Hospital care for children. Guidelines for the management of common childhood illnesses. Second Edition, 2013. Available: https://apps.who.int/iris/bitstream/handle/10665/81170/9789241548373_eng.pdf?sequence=1 [Accessed 28 Dec 2021]

12. A Paediatric Handbook for Malawi. Third Edition, 2008. Available: http://cms.kcn.unima.mw:8002/moodle/file.php/1/Phillips_Kazembe_Paediatric_Handbook_for_Malawi_2008.pdf [Accessed 28 Dec 2021]

13. National Malaria Control Programme, Ministry of Health, Government of Malawi. Guidelines for the Treatment of Malaria in Malawi. 4th Edition, 2013. Available: https://www.severemalaria.org/sites/mmv-smo/files/content/attachments/2017-03-03/Malawi%202013%20Edition%20of%20Malaria%20Treatment%20Guidelines.pdf [Accessed 28 Dec 2021]

14. Ministry of Health. Malawi Standard Treatment Guidelines. 5th Edition, 2015. Available: https://extranet.who.int/ncdccs/Data/MWI_D1_Malawi-Standard-Treatment-Guidelines-Essential-Medicines-List-2015.pdf [Accessed 28 Dec 2021]



Confidential: For Review Only

15. Liu L, Oza S, Hogan D, et al. Global, regional, and national causes of under-5 mortality in 2000-15: an updated systematic analysis with implications for the Sustainable Development Goals. *Lancet* 2016;**388**:3027-3035.

16. Agweyu A, Lilford RJ, English M. Appropriateness of clinical severity classification of new WHO childhood pneumonia guidance: a multi-hospital, retrospective, cohort study. *Lancet Glob Health* 2018;**6**:e74-e83.

17. Berkley JA, Ross A, Mwangi I, et al. Prognostic indicators of early and late death in children admitted to district hospital in Kenya: cohort study. *BMJ*. 2003;**326**:361.

18. Rees CA, Hooli S, King C, et al. External validation of the RISC, RISC-Malawi, and PERCH clinical prediction rules to identify risk of death in children hospitalized with pneumonia. *J Glob Health* 2021;**11**:04062.

19. Graham H, Bakare AA, Ayede AI, et al. Diagnosis of pneumonia and malaria in Nigerian hospitals: A prospective cohort study. *Pediatr Pulmonol* 2020;**55** Suppl 1:S37-s50.

20. Sigaúque B, Roca A, Bassat Q, et al. Severe pneumonia in Mozambican young children: clinical and radiological characteristics and risk factors. *J Trop Pediatr* 2009;**55**:379-87.

21. Källander K, Nsungwa-Sabiiti J, Peterson S. Symptom overlap for malaria and pneumonia--policy implications for home management strategies. *Acta Trop* 2004;**90**:211-4.

22. Mwaniki MK, Nokes DJ, Ignas J, et al. Emergency triage assessment for hypoxaemia in neonates and young children in a Kenyan hospital: an observational study. *Bull World Health Organ* 2009;**87**:263-70.



Confidential: For Review Only

23. Mogensen CB, Sørensen JE, Bjorkman A. Pallor as a sign of anaemia in small Tanzanian children at different health care levels. *Acta Trop* 2006;**99**:113-8.

24. Kalter HD, Burnham G, Kolstad PR, et al. Evaluation of clinical signs to diagnose anaemia in Uganda and Bangladesh, in areas with and without malaria. *Bull World Health Organ* 1997;**75** Suppl 1:103-11.

25. Hooli S, King C, Zadutsa B, et al. The Epidemiology of Hypoxemic Pneumonia among Young Infants in Malawi. Am J Trop Med Hyg 2020;**102**:676-83.

26. Hooli S, Colbourn T, Lufesi N, et al. Predicting Hospitalised Paediatric Pneumonia Mortality Risk: An External Validation of RISC and mRISC, and Local Tool Development (RISC-Malawi) from Malawi. *PLoS One* 2016;**11**:e0168126.

27. Mayxay M, Pukrittayakamee S, Chotivanich K, Looareesuwan S, White NJ. Persistence of Plasmodium falciparum HRP-2 in successfully treated acute falciparum malaria. *Trans R Soc Trop Med Hyg* 2001;**95**:179-82.

Confidential: For Review Only

457 Table 1. Association between hospitalization characteristics and outcome among
458 children 1-59 months old with WHO-defined severe pneumonia without HIV, severe
459 malnutrition, and/or hypoxemia in an observational study
460

| Characteristic | | Survived N=863 | Died N=21 | p-value |
|---|---|---|---|---|
| Demographics | | | | |
| Age in months, median (IQR)[1] | | 9.2 (4.7, 17.3) | 8.1 (6.5, 12.0) | 0.74 |
| Sex, n (%)[2] | Females | 353 (97.5%) | 9 (2.5%) | 0.86 |
| | Males | 510 (97.7%) | 12 (2.3%) | |
| Clinical features | | | | |
| Respiratory rate (breaths/min), mean (SD)[4] | | 59.6 (12.9) | 60.3 (13.7) | 0.82 |
| Fast breathing for age, n (%)[2] | Yes | 720 (97.6%) | 18 (2.4%) | 0.78 |
| | No | 143 (97.9%) | 3 (2.1%) | |
| Pulse rate (beats/min), mean (SD)[4] | | 162.2 (20.4) | 170.0 (30.4) | 0.09 |
| Pulse rate >180 beats/min, n (%)[2] | Yes | 113 (95.0%) | 6 (5.0%) | 0.14 |
| | No | 400 (97.6%) | 10 (2.4%) | |
| Pulse rate >160 beats/min, n (%)[2] | Yes | 165 (97.6%) | 4 (2.4%) | 0.15 |
| | No | 184 (99.5%) | 1 (0.5%) | |
| Severe chest indrawing, n (%)[2] | Yes | 760 (97.8%) | 17 (2.2%) | 0.32 |
| | No | 103 (96.3%) | 4 (3.7%) | |
| Head nodding, n (%)[2] | Yes | 180 (95.7%) | 8 (4.3%) | 0.06 |
| | No | 683 (98.1%) | 13 (1.9%) | |
| Grunting, n (%)[2] | Yes | 102 (91.1%) | 10 (8.9%) | <0.01 |
| | No | 760 (98.6%) | 11 (1.4%) | |
| Stridor when calm, n (%)[2] | Yes | 12 (85.7%) | 2 (14.3%) | <0.01 |
| | No | 851 (97.8%) | 19 (2.2%) | |
| Apnea, n (%)[2] | Yes | 6 (85.7%) | 1 (14.3%) | 0.04 |
| | No | 857 (97.7%) | 20 (2.3%) | |
| Nasal flaring, n (%)[2] | Yes | 507 (97.6%) | 12 (2.3%) | 0.88 |
| | No | 356 (97.5%) | 9 (2.5%) | |
| Measurements and laboratory results | | | | |
| MUAC in cm, mean (SD)[3] | | 14.0 (1.2) | 13.3 (1.0) | <0.01 |
| MUAC 11.5-13.5 cm, n (%)[2] | Yes | 255 (96.2%) | 10 (3.8%) | 0.07 |
| | No | 608 (98.2%) | 11 (1.8%) | |
| Hb (g/dL), mean (SD)[3] | | 10.0 (2.0) | 7.8 (3.0) | <0.01 |
| Hb <5 g/dL[2] | Yes | 24 (88.9%) | 3 (11.1%) | <0.01 |
| | No | 839 (99.6%) | 18 (2.%) | |
| Malaria positive, n (%)[4] | Yes | 193 (94.6%) | 11 (5.4%) | <0.01 |
| | No | 663 (98.5%) | 10 (1.5%) | |
| $SpO_2$ (%), median (IQR)[1] | | 96 (95, 98) | 96 (94, 98) | 0.49 |

Confidential: For Review Only

| | | | | |
|---|---|---|---|---|
| $SpO_2$[2] | 93-100% | 787 (97.8%) | 18 (2.2%) | 0.38 |
| | 90-92% | 76 (96.2%) | 3 (3.7%) | |

WHO indicates World Health Organization; IQR, interquartile range; SD, standard deviation; MUAC, mid-upper arm circumference; Hb, hemoglobin.
[1]Wilcoxon rank sum
[2]Fisher's exact
[3]Two sample t-test
[4]7 participants were missing test results

Confidential: For Review Only

469 Table 2: Association between mortality and number of risk factors (apnea, stridor when
470 calm, grunting, hemoglobin < 5 g/dL, malaria test positive)
471

| Number of risk factors[1] | Odds ratio (95% CI) | p-value |
|---|---|---|
| 1 | Referent | – |
| 2 | 4.6 (1.5, 14.3) | <0.01 |
| 3 | 11.6 (3.2, 41.2) | <0.01 |
| 4 | 35.4 (7.4, 168.8) | <0.01 |

472 [1]No child had all 5 risk factors
473

Confidential: For Review Only

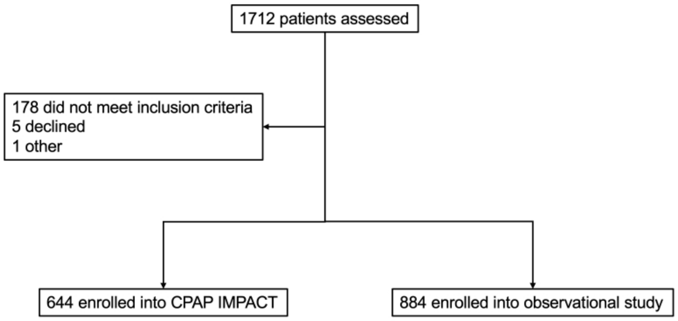


1649x1165mm (72 x 72 DPI)

Supplemental Materials

Supplemental Table 1: Sensitivity analysis of survival and death, excluding children with no malaria result

| Factor | | Survived | Died | p-value |
|---|---|---|---|---|
| N=877 | | 856 | 21 | |
| Sex, n (%) | Males | 505 (59.0%) | 12 (57.1%) | 1.00 |
| | Females | 351 (41.0%) | 9 (42.9%) | |
| Age in months, median (IQR) | | 9.2 (4.6, 17.2) | 8.1 (6.5, 12.0) | 0.76 |
| Respiratory rate (breaths/min), mean (SD) | | 59.6 (12.9) | 60.3 (13.7) | 0.82 |
| Fast breathing for age | No | 143 (16.7%) | 3 (14.3%) | 1.00 |
| | Yes | 713 (83.3%) | 18 (85.7%) | |
| Pulse rate (beats/min), mean (SD) | | 162.3 (20.4) | 170.0 (30.4) | 0.09 |
| Pulse rate >180 beats/min, n (%) | No | 397 (77.8%) | 10 (62.5%) | 0.22 |
| | Yes | 113 (22.2%) | 6 (37.5%) | |
| Pulse rate >160 beats/min, n (%) | No | 181 (52.5%) | 1 (20.0%) | 0.20 |
| | Yes | 164 (47.5%) | 4 (80.0%) | |
| Severe chest indrawing, n (%) | No | 99 (11.6%) | 4 (19.0%) | 0.30 |
| | Yes | 757 (88.4%) | 17 (81.0%) | |
| Head nodding, n (%) | No | 676 (79.0%) | 13 (61.9%) | 0.10 |
| | Yes | 180 (21.0%) | 8 (38.1%) | |
| Grunting, n (%) | No | 754 (88.2%) | 11 (52.4%) | 0.0 |
| | Yes | 101 (11.8%) | 10 (47.6%) | |
| Stridor when calm, n (%) | No | 844 (98.6%) | 19 (90.5%) | 0.04 |
| | Yes | 12 (1.4%) | 2 (9.5%) | |
| Apnea, n (%) | No | 850 (99.3%) | 20 (95.2%) | 0.16 |
| | Yes | 6 (0.7%) | 1 (4.8%) | |
| Nasal flaring, n (%) | No | 350 (40.9%) | 9 (42.9%) | 1.00 |
| | Yes | 506 (59.1%) | 12 (57.1%) | |
| MUAC in cm, mean (SD) | | 14.0 (1.2) | 13.3 (1.0) | <0.01 |
| MUAC 11.5-13.5 cm, n (%) | No | 604 (70.6%) | 11 (52.4%) | 0.09 |
| | Yes | 252 (29.4%) | 10 (47.6%) | |
| Hb (g/dL), mean (SD) | | 10.0 (2.0) | 7.8 (3.0) | <0.01 |
| Hb < 5 g/dL | No | 832 (97.2%) | 18 (85.7%) | 0.02 |
| | Yes | 24 (2.8%) | 3 (14.3%) | |
| SpO2 (%), median (IQR) | | 96.0 (95.0, 98.0) | 96.0 (94.0, 98.0) | 0.49 |

Confidential: For Review Only

| | | | | |
|---|---|---|---|---|
| SpO2 93-100% | 0 | 781 (91.2%) | 18 (85.7%) | 0.42 |
| SpO2 90-92% | 1 | 75 (8.8%) | 3 (14.3%) | |
| Malaria positive, n (%) | No | 663 (77.5%) | 10 (47.6%) | 0.003 |
| | Yes | 193 (22.5%) | 11 (52.4%) | |

Confidential: For Review Only

Supplemental Table 2. Characteristics of children 1-59 months old with WHO-defined severe pneumonia at Salima District Hospital, Malawi in an observational study compared to a randomized controlled trial CPAP IMPACT

| Characteristic | Observational study N=884 | CPAP IMPACT N=644 | p-value |
|---|---|---|---|
| Age in months, median (IQR) | 9.2 (4.7, 17.2) | 7.7 (3.1, 15.2) | <0.01 |
| Females, n (%) | 362 (41.0%) | 299 (46.4%) | 0.03 |
| Males, n (%) | 522 (59.0%) | 345 (53.6%) | |
| Weight in kg, mean (SD) | 8.5 (2.5) | 7.2 (2.8) | <0.01 |
| MUAC in cm, mean (SD) | 14.0 (1.2) | 12.7 (1.9) | <0.01 |
| HIV-infected, n (%) | 0 (0.0%) | 36 (5.6%) | <0.01 |
| HIV-exposed uninfected, n (%) | 0 (0.0%) | 99 (15.4%) | <0.01 |
| Severe malnutrition, n (%) | 0 (0.0%) | 222 (34.5%) | <0.01 |
| Hypoxemia only, n (%) | 0 (0.0%) | 287 (44.6%) | <0.01 |
| Pulse rate in beats/min, mean (SD) | 162.4 (20.7) | 164.1 (24.0) | 0.15 |
| Respiratory rate in breaths/min, mean (SD) | 59.6 (12.9) | 61.9 (15.1) | <0.01 |
| $SpO_2$, median (IQR) | 96 (95, 98) | 88 (83, 94) | <0.01 |
| $SpO_2$ 93-100%, n (%) | 805 (91.1%) | 191 (29.7%) | |
| $SpO_2$ 90-92%, n (%) | 79 (8.9%) | 38 (5.9%) | |
| $SpO_2$ <90%, n (%) | 0 (0.0%) | 415 (64.4%) | |
| 1 or more respiratory danger signs[1], n (%) | 866 (98.0%) | 638 (99.1%) | 0.09 |
| Severe chest indrawing, n (%) | 777 (87.9%) | 575 (89.3%) | 0.41 |
| Head nodding, n (%) | 188 (21.3%) | 176 (27.3%) | <0.01 |
| Grunting, n (%) | 112 (12.7%) | 154 (23.9%) | <0.01 |
| Very fast breathing for age, n (%)[2] | 256 (29.0%) | 211 (32.8%) | 0.11 |
| Stridor when calm, n (%) | 14 (1.6%) | 13 (2.0%) | 0.52 |
| Apnea, n (%) | 7 (0.8%) | 37 (5.7%) | <0.01 |
| Nasal flaring, n (%) | 519 (58.7%) | 471 (73.1%) | <0.01 |
| Convulsions, n (%) | 45 (5.1%) | 61 (9.5%) | <0.01 |
| Inability to feed, n (%) | 27 (3.1%) | 78 (12.1%) | <0.01 |
| Vomiting everything, n (%) | 12 (1.4%) | 19 (3.0%) | 0.03 |
| Blantyre Coma Score ≤4, n (%) | 20 (2.3%) | 55 (8.5%) | <0.01 |
| Crackles on auscultation, n (%) | 429 (48.5%) | 311 (48.3%) | 0.95 |
| Wheeze on auscultation, n (%) | 160 (18.1%) | 128 (19.9%) | 0.38 |
| Hb <5 g/dL, n (%) | 27 (3.1%) | 50 (7.8%) | <0.01 |
| Hb (g/dL), mean (SD) | 10.0 (2.1) | 9.5 (2.6) | <0.01 |
| Malaria positive, n (%)[3] | 204 (23.3%) | 197 (30.6%) | <0.01 |
| Death, n (%) | 21 (2.4%) | 88 (13.7%) | <0.01 |

WHO indicates World Health Organization; IQR, interquartile range; SD, standard deviation; MUAC, mid-upper arm circumference; $SpO_2$; peripheral oxyhemoglobin saturation; Hb, hemoglobin.

[1]Any of the following signs of respiratory distress: severe chest indrawing, head nodding, grunting, very fast breathing for age, stridor when calm, apnea, nasal flaring

[2]≥80 breaths/min if 30-59 days old, ≥70 breaths/min if 2-11 months old, and ≥60 breaths/min if 12-59 months old
[3]Rapid test positive, 10 observational study participants were missing test results

Confidential: For Review Only

BMJ Paediatrics Open

# Identifying modifiable risk factors for mortality in children aged 1-59 months admitted with WHO-defined severe pneumonia: a single centre observational cohort study from rural Malawi

| | |
|---|---|
| Journal: | *BMJ Paediatrics Open* |
| Manuscript ID | bmjpo-2021-001330.R2 |
| Article Type: | Original research |
| Date Submitted by the Author: | 16-Mar-2022 |
| Complete List of Authors: | Eckerle, Michelle; Cincinnati Children's Hospital Medical Center, Pediatrics<br>Mvalo, Tisungane; University of North Carolina Project; University of North Carolina at Chapel Hill School of Medicine, Department of Pediatrics<br>Smith, Andrew; The University of Utah School of Medicine<br>Kondowe, Davie; University of North Carolina Project<br>Makonokaya, Don; University of North Carolina Project<br>Vaidya, Dhananjay; Johns Hopkins University School of Medicine<br>Hosseinipour, Mina; University of North Carolina Project; University of North Carolina at Chapel Hill School of Medicine<br>McCollum, Eric D.; Johns Hopkins School of Medicine, Department of Paediatrics |
| Keywords: | Epidemiology, Health services research, Mortality |
| | |

SCHOLARONE™
Manuscripts

*I, the Submitting Author has the right to grant and does grant on behalf of all authors of the Work (as defined in the below author licence), an exclusive licence and/or a non-exclusive licence for contributions from authors who are: i) UK Crown employees; ii) where BMJ has agreed a CC-BY licence shall apply, and/or iii) in accordance with the terms applicable for US Federal Government officers or employees acting as part of their official duties; on a worldwide, perpetual, irrevocable, royalty-free basis to BMJ Publishing Group Ltd ("BMJ") its licensees and where the relevant Journal is co-owned by BMJ to the co-owners of the Journal, to publish the Work in this journal and any other BMJ products and to exploit all rights, as set out in our licence.*

*The Submitting Author accepts and understands that any supply made under these terms is made by BMJ to the Submitting Author unless you are acting as an employee on behalf of your employer or a postgraduate student of an affiliated institution which is paying any applicable article publishing charge ("APC") for Open Access articles. Where the Submitting Author wishes to make the Work available on an Open Access basis (and intends to pay the relevant APC), the terms of reuse of such Open Access shall be governed by a Creative Commons licence – details of these licences and which Creative Commons licence will apply to this Work are set out in our licence referred to above.*

*Other than as permitted in any relevant BMJ Author's Self Archiving Policies, I confirm this Work has not been accepted for publication elsewhere, is not being considered for publication elsewhere and does not duplicate material already published. I confirm all authors consent to publication of this Work and authorise the granting of this licence.*

Confidential: For Review Only

Identifying modifiable risk factors for mortality in children aged 1-59 months admitted with WHO-defined severe pneumonia: a single centre observational cohort study from rural Malawi

Michelle Eckerle, MD[1,2]
Tisungane Mvalo, FC Paed[3,4]
Andrew G. Smith, MD[5]
Davie Kondowe, BBiomedSc[3]
Don Makonokaya, BScPH[3]
Dhananjay Vaidya, PhD[6]
Mina C. Hosseinipour, MD[3,8]
Eric D. McCollum, MD, MPH[9,10]

[1]Division of Emergency Medicine, Cincinnati Children's Hospital Medical Center, Cincinnati, Ohio, USA
[2]Department of Pediatrics, University of Cincinnati College of Medicine, Cincinnati, Ohio, USA
[3]University of North Carolina Project Malawi, Lilongwe, Malawi
[4]Department of Pediatrics, School of Medicine, University of North Carolina at Chapel Hill, NC, USA.
[5]Department of Pediatrics, University of Utah School of Medicine, Salt Lake City, Utah, USA

Confidential: For Review Only

[6]Department of Medicine, Epidemiology and the BEAD Core, Johns Hopkins University, Baltimore, MD, USA

[7]Department of Pediatrics, BEAD Core, Johns Hopkins University, Baltimore, MD, USA

[8]Division of Infectious Disease, University of North Carolina at Chapel Hill, Chapel Hill, North Carolina, USA

[9]Global Program for Pediatric Respiratory Sciences, Eudowood Division of Pediatric Respiratory Sciences, Department of Pediatrics, School of Medicine, Johns Hopkins University, Baltimore, Maryland, United States

[10]Department of International Health, Bloomberg School of Public Health, Johns Hopkins University, Baltimore, Maryland, United States

Corresponding author:

Eric D. McCollum, MD, MPH

Email: emccoll3@jhmi.edu

Word count: 2,421

Confidential: For Review Only

Abstract:

Objective: Although HIV-infection, severe malnutrition, and hypoxemia are associated with high mortality in children with World Health Organization (WHO)-defined severe pneumonia in sub-Saharan Africa, many do not have these conditions and yet mortality remains elevated compared to high-resource settings. Further stratifying mortality risk for children without these conditions could permit more strategic resource utilization and improved outcomes. We therefore evaluated associations between mortality and clinical characteristics not currently recognized by the WHO as high-risk among children in Malawi with severe pneumonia but without HIV (including exposure), severe malnutrition, and hypoxemia.

Methods: Between May 2016 and March 2018 we conducted a prospective observational study alongside a randomized controlled trial (CPAP IMPACT) at Salima District Hospital in Malawi. Children 1-59 months hospitalized with WHO-defined severe pneumonia without severe malnutrition, HIV, and hypoxemia were enrolled. Study staff assessed children at admission and ascertained hospital outcomes. We compared group characteristics using Student t, rank sum, Chi-Square or Fisher's Exact tests as appropriate.

Results: Among 884 participants grunting (10/112 (8.9%) vs 11/771 (1.4%)), stridor (2/14 (14.2%) vs 19/870 (2.1%)), hemoglobin <5 g/dL (3/27 (11.1%) vs 18/857 (2.1%)), and malaria (11/204 (5.3%) vs 10/673 (1.4%)) were associated with mortality compared to children without these characteristics. Children who survived had a 2.2 g/dL higher



Confidential: For Review Only

mean hemoglobin and 0.7 cm higher mean mid-upper arm circumference (MUAC) than those who died.

Conclusion: In this single-center study, our analysis identifies potentially modifiable risk factors for mortality among hospitalized Malawian children with severe pneumonia: specific signs of respiratory distress (grunting, stridor), hemoglobin <5 g/dL, and malaria infection. Significant differences in mean hemoglobin and MUAC were observed between those who survived and those who died. These factors could further stratify mortality risk among hospitalized Malawian children with severe pneumonia lacking recognized high-risk conditions.

Confidential: For Review Only

What is known about this subject

- Lower respiratory infections like pneumonia kill an estimated 800,000 children annually, making it the number one infectious cause of pediatric deaths globally.
- Among children with pneumonia, important drivers of mortality in low-income and middle-income countries (LMICs) are severe malnutrition, HIV infection or exposure, and hypoxemia.
- Children in LMICs with severe pneumonia but without HIV (including exposure), severe malnutrition, and hypoxemia may account for a large proportion of the overall pneumonia burden and mortality but data is limited.

What this study adds

- About 20% of pneumonia deaths at one rural district hospital in Malawi over two years were attributable to children lacking HIV, severe malnutrition, and hypoxemia.
- Among Malawian children with severe pneumonia without HIV, severe malnutrition, and hypoxemia, respiratory distress (stridor, grunting), hemoglobin <5 g/dL, and malaria predicted mortality.
- Significant differences in mean hemoglobin and mid-upper arm circumference were present in severe pneumonia cases lacking recognized high-risk conditions who survived or died.

Confidential: For Review Only

## Introduction

Pneumonia causes approximately 800,000 deaths outside of the neonatal period among under 5 year olds worldwide and this burden is disproportionately borne in low-income and middle-income countries (LMICs).[1] Severe malnutrition, HIV-infection or exposure, and hypoxemia (peripheral oxyhemoglobin saturation ($SpO_2$) <90%) place children with pneumonia at higher risk of death.[1-4] While children with severe pneumonia but without these conditions may have relatively lower mortality, they still may comprise a substantial overall burden of pneumonia morbidity and mortality in LMICs.[1] For example, in Malawi approximately half of child pneumonia deaths were attributable to HIV and almost 90% of hospitalized pneumonia were among children without hypoxemia.[4,5] In a multi-country severe pneumonia study 61% of deaths occurred among children without severe malnutrition.[6] Identifying clinical characteristics that predict mortality, can be objectively measured, and may be modifiable among pediatric pneumonia cases lacking severe malnutrition, HIV (including exposure), and hypoxemia could represent an effective strategy for lowering pneumonia deaths in LMICs.

The World Health Organization (WHO) recommends a syndromic approach to child pneumonia diagnosis and management in LMICs to improve implementation feasibility.[7] Recommended treatment for WHO-defined severe pneumonia includes hospitalization, parenteral antibiotics, and oxygen.[7] Understanding which children with WHO-defined severe pneumonia in LMICs are at high risk of death is important as it can impact resource allocation in austere settings where advanced care and other resources are limited.[8]



Confidential: For Review Only

In conjunction with a randomized controlled trial called CPAP IMPACT, which studied children with WHO-defined severe pneumonia and at least one high-risk condition (severe malnutrition, HIV-infection or exposure, and/or hypoxemia),[9,10] we conducted a parallel prospective observational study of children with WHO-defined severe pneumonia but without any of these three high-risk conditions. Our main objectives were to contextualize observational study participants through their clinical characteristics and outcomes, and then to identify clinical predictors of mortality with a priority towards factors that may be objectively measured, potentially modifiable, and not included in WHO pneumonia guidelines.

## Methods

We conducted a pre-specified sub-study in conjunction with a randomized controlled trial (CPAP IMPACT).[9,10] CPAP IMPACT compared bubble continuous positive airway pressure (bCPAP) to low flow oxygen for children with WHO-defined severe pneumonia and at least one high-risk condition. From May 2016 through March 2018, children with WHO-defined severe pneumonia and ineligible for CPAP IMPACT were invited to participate in this ancillary prospective observational study. The study took place at Salima District Hospital in Salima, Malawi, a secondary hospital lacking intensive care resources. Institutional review boards approved the protocol. Enrolled participants provided written consent.

Confidential: For Review Only

During the study period children admitted to Salima District Hospital were screened for eligibility at all times. Children were eligible if 1-59 months with WHO-defined severe pneumonia and without any of three high-risk conditions: (1) HIV-infection (<12 months old with a positive HIV DNA PCR or ≥12 months old with HIV antibodies) or exposure (<24 months old born to a HIV-infected mother and not meeting HIV-infection criteria), (2) severe malnutrition (weight for age z score < -3 standard deviations (SD), mid-upper arm circumference (MUAC) <11.5 cm, and/or bilateral pedal edema), or (3) a $SpO_2$ <90%.

Per WHO guidelines, children could meet severe pneumonia criteria by having cough and/or difficult breathing plus signs of pneumonia with at least one general danger sign or signs of respiratory distress with or without a general danger sign.[11] Respiratory distress included head nodding, nasal flaring, grunting, stridor when calm, tracheal tugging, severe chest indrawing, very fast breathing for age (≥80 breaths/min if 30-59 days old, ≥70 breaths/min if 2-11 months old, and ≥60 breaths/min if 12-59 months old), or apnea. Signs of pneumonia included fast breathing (60-79 breaths/min if 30-59 days old, 50-69 breaths/min if 2-11 months old, and 40-59 breaths/min if 12-59 months old) and/or chest indrawing. Inability to feed, vomiting everything, lethargic or unconscious (Blantyre Coma Score ≤4),[12] and convulsions comprised general danger signs.

Consistent with national guidelines malaria infection was confirmed on all participants at enrollment using rapid antigen testing (SD Bioline (HRP2/pLDH, sensitivity 97.4%-99.7%, specificity 99.3%-99.7%)) as routine microscopy was limited.[13] Anemia was also

Confidential: For Review Only

assessed by point-of-care testing (Hemocue 301+). A Rad5 pulse oximeter (Masimo, Irvine, CA, USA) measured the $SpO_2$ on children breathing in room air. Study staff recommended initial evaluations and treatments at enrollment and thereafter care was led by government hospital staff, including oxygen, antibiotics, and fluids. According to local guidelines, all patients received benzylpenicillin and gentamicin and could be switched to ceftriaxone for treatment failure.[14] Similarly, per local guidelines, severe malaria was treated with artesunate and uncomplicated malaria by lumefantrine-artemether.[13] Children were followed passively by study staff until hospital outcome. This study's primary outcome was in-hospital mortality.

*Statistical Analysis*

Bivariable analysis comparing clinical variables with in-hospital mortality was performed as follows: The Student's *t*-test was used to assess differences between the means of normally distributed continuous variables (tabulated as means and standard deviations (SDs)), the non-parametric Mann-Whitney U test evaluated differences in the rank sums of continuous variables (tabulated as medians and interquartile range (IQR) presented as 25$^{th}$ and 75$^{th}$ percentile, Chi Square and Fisher's Exact tests (if the large sample assumption could not be justified) analyzed categorical clinical variables (tabulated as numbers and percentages). To understand whether an association existed between mortality and the number of significant risk factors per patient, logistic regression was performed. Although we planned to fit multivariable regression models for mortality we determined it was not advisable due to the number of mortality events experienced. All analyses were conducted using Stata (version 15.1; StataCorp, College Station, Texas).



Confidential: For Review Only

## Patient and public involvement

Patients and the public in Malawi were sensitized to the development, design, recruitment and conduct via local meetings before, during, and after the research.

## Results

From May 2016 enrollment began for the observational study and concluded at the same time as the parent CPAP IMPACT trial in March 2018 (Figure 1). Among 1,062 children that did not meet inclusion criteria for CPAP IMPACT, 884 met criteria for the observational study and agreed to participate.

We sought to identify whether any participant characteristics – with an emphasis on objective and potentially modifiable factors not included in the WHO pneumonia guidelines[7] – had associations with hospital mortality (Table 1). Children with respiratory distress signs like grunting (10/112 (8.9%) vs 11/771 (1.4%), p<0.01) and stridor when calm (2/14 (14.2%) vs 19/870 (2.1%), p=0.04) had higher mortality rates than children without these signs. Anemia and malaria were also associated with mortality. Specifically, children who died, compared to survivors, had a 2.2 g/dL lower mean hemoglobin (7.8 g/dL [SD 3.0] vs 10.0 g/dL [SD 2.0], p<0.01). Children with a hemoglobin <5 mg/dL also had higher mortality than those with a 5 mg/dL or higher measurement (11.1% vs 2.1%, p<=.02). Children positive for malaria had a mortality of 5.4% while children testing negative had a mortality rate of 1.4% (p<0.01). Children with malaria, compared to those testing negative, had a 1.9 mg/dL lower mean hemoglobin





Confidential: For Review Only

level (8.5 g/dL vs 10.4 g/dL, $p<0.01$). In a sensitivity analysis excluding seven children without a malaria test result, the overall findings were consistent (Supplemental Table 1).

Participants who died had a 1.7 cm lower mean MUAC than those who did not die ($p<0.01$). Notably, there was no difference between those who survived and those who did not based on the admission $SpO_2$ distribution ($p=0.49$), and although the point estimates for mortality were higher among children with a $SpO_2$ 90-92%, compared to 93-100%, these did not reach statistical significance (3.7% vs 2.2%, $p=0.42$).

Of the identified risk factors for mortality, we considered dichotomous factors (stridor when calm, grunting, malaria status, hemoglobin <5 g/dL) and used logistic regression to determine whether the odds of mortality increased with increasing numbers of risk factors per patient. We found that compared to children with only one risk factor, children with two, three, or four risk factors had an increased odds ratio for death that increased with an increasing number of risk factors (Table 2).

In Supplemental Table 2 we compared observational participants to children enrolled in CPAP IMPACT to provide context for both groups as well as the spectrum of pneumonia at Salima District Hospital during the study period. The mortality of observational children was 11.3 absolute percentage points lower than those in CPAP IMPACT (2.4% vs 13.7%, $p<0.01$). Altogether, when accounting for trial participants and

Confidential: For Review Only

the observational group, the hospital mortality of severe pneumonia was 7.1% (109/1,528).

Discussion:

We identified potentially modifiable, objectively measurable risk factors for mortality among an observational cohort of Malawian children with WHO-defined severe pneumonia and without severe malnutrition, HIV, or hypoxemia. We found those children who died were more likely to present with respiratory distress, anemia, malaria, and a lower MUAC. Our results also show that during the study period severe pneumonia was generally as frequent among Malawian children without high risk conditions as they were with them, and that about 20% of pneumonia deaths at Salima District Hospital during this period were attributable to lower risk children. Assuming these data are representative then potentially high numbers of pneumonia deaths among sub-Saharan African children without severe malnutrition, HIV, and hypoxemia may occur each year.[15] Altogether, if confirmed, our results imply such children could be important in the broader pneumonia epidemiological context and may benefit from more systematic scrutiny of these additional, potentially modifiable domains of risk, (1) respiratory distress, (2) anemia, (3) malaria, and, (4) non-severe malnutrition, through more rigorous screening and testing.

Currently, only MUAC is routinely recommended by the WHO IMCI guidelines for use at frontline health facilities and hospital outpatient clinics serving as an entry point for most patients.[7] Instead, guidelines recommend using caregiver report to identify difficult

Confidential: For Review Only

breathing, checking for anemia by examining for pallor, and testing for malaria if fever is present (in endemic areas).[7] While severe malnutrition, HIV, and hypoxemia confer a higher mortality risk, there is value in identifying specific features that convey a higher probability of death among lower risk but severely ill patients. It is possible these children, if appropriately identified, may be more treatment responsive and more likely to survive; the ability to target care would be beneficial. For example, such children may be more successfully managed if cohorted into hospital units with higher nurse to patient ratios that allow closer monitoring and more meticulous care. Results from CPAP IMPACT suggest meaningful reductions in mortality among higher risk children with severe pneumonia may be challenging to achieve with available approaches in LMICs.

Previous studies reported similar findings to this study, though analyses aggregate children with and without high-risk conditions or severe and non-severe pneumonia. A large meta-analysis of children from LMICs found that young age (<2 months), *Pneumocystis jirovecii* infection, chronic underlying diseases, HIV/AIDS and severe malnutrition all increased the likelihood of death.[2] Within sub-Saharan Africa, a Kenyan study of pediatric pneumonia hospitalizations sought to establish mortality rates and risk factors among children with pneumonia.[16] Among those with non-severe disease, pallor and a weight-for-age z-score <3 SD were associated with death, but having respirations ≥70 breaths/minute, moderate malnutrition, moderate dehydration, or admission in a malaria-endemic region also increased mortality risk. Another Kenyan study of hospitalized children found overall mortality was 5%, and severe anemia, jaundice,



https://mc.manuscriptcentral.com/bmjpo

Confidential: For Review Only

chest indrawing, deep breathing, neurologic status, and axillary temperature (>39ºC or <36ºC) were predictive of immediate death.[17]

In our observational cohort children who died were more likely to have a lower hemoglobin and were also more likely to have malaria than those who survived. As expected, a significant difference in mean hemoglobin existed between children with a positive versus negative malaria test. Given the syndromic approach to disease used by healthcare workers in LMICs, an overlap between primary respiratory illness and malaria has been previously reported. In a prospective cohort of children in Nigerian hospitals, 31% of those with pneumonia also had malaria,[18] while in Mozambique 19% of children with severe pneumonia had malaria.[19] In Uganda, a study of children attending 14 health centers found 30% had both pneumonia and malaria.[20] A large Kenyan cohort of hospitalized hypoxemic children found a final diagnosis of malaria more frequently than lower respiratory tract infection (35% versus 32%).[21]

Unfortunately, clinical pallor has been shown as unreliable for detecting anemia. A study of hospitalized Tanzanian children found 8% sensitivity and 98% specificity for pallor identifying severe anemia.[22] Among children in Uganda and Bangladesh severe palmar and conjunctival pallor had sensitivities between 10%-50% and a specificity of 99% for severe anemia.[23] Altogether, given the co-incidence of these conditions, the mortality risk associated with severe malaria and anemia, and the limited performance of pallor, routine hemoglobin and malaria testing among children with difficulty breathing merits further consideration.





Confidential: For Review Only

Unlike this study, prior inpatient pediatric studies from Malawi found associations between moderately low $SpO_2$ ranges and increased odds of mortality among children with pneumonia (between 50% and 330% higher).[24,25] The pediatric populations in these studies differed as they included severe malnutrition and/or HIV cases. Although in our study we also observed higher point estimates for mortality among children with a $SpO_2$ 90-92%, compared to 93-100%, this association did not reach statistical significance. We expect that with a larger sample size a similar association between $SpO_2$ and mortality would have been observed in this study.

There are several limitations. First, malaria detection was determined by rapid antigen testing rather than peripheral blood microscopy. While children in endemic settings may have a positive rapid test due to persistent antigenemia rather than an acute infection,[26] our use of rapid testing was consistent with locally recommended practice in Malawi, a setting with limited microscopy capacity.[13] Second, the cohort experienced 21 deaths, and this restricted our ability to fit multivariable models for mortality as planned. Third, although our prospective design and population of rural Malawian children at a district hospital should be considered strengths, this is a single center study. Increasing the certainty of our findings remains important and a larger, multi-center study is needed.

In sum, we identified multiple risk factors for death (specific signs of respiratory distress (stridor, grunting), hemoglobin <5 g/dL, and malaria) among Malawian children with severe pneumonia but without severe malnutrition, HIV infection or exposure, and



Confidential: For Review Only

hypoxemia. Children who died had a lower MUAC than those who survived, but statistically significant thresholds were not apparent. Based on these findings systematic screening of children with severe pneumonia for additional signs of respiratory distress, checking of hemoglobin levels, testing for malaria (when endemic), and measuring MUAC, followed by closer monitoring and standard treatment for any identified abnormalities, seem feasible and warrant further consideration. Intervention studies examining the modifiability of these risk factors in similar populations are needed as is larger, confirmatory observational research in other LMIC settings.

Figure 1. Study Profile

Acknowledgements:

We thank the children and their caregivers who participated, Salima District Hospital and the Malawi Ministry of Health for their support of this research, and the entire CPAP IMPACT study team for their dedication and care of the children in this study. We also acknowledge the important contributions of Dr. Veena Billioux to this work.

Author contributions:

EDM acquired the funding. ME and EDM were responsible for conceptualization and design. EDM curated the data. DK and DM collected the data. DV, VB, ME, and EDM were responsible for data analysis. ME, EDM, TM, AGS, and MH were responsible for data interpretation. ME and EDM wrote the original draft. ME, EDM, TM, AGS, DK, DM, DV, and MH were responsible for writing, review, and editing. EDM is the guarantor.



Confidential: For Review Only

Competing Interests Statement:

We declare no competing interests.

Funding statement:

The Bill & Melinda Gates Foundation (OPP1123419), International AIDS Society (141022) and Health Empowering Humanity (award number not applicable) provided funding for this work.

Data availability statement:

Data are available upon reasonable request. A de-identified dataset and data dictionary will be provided after a signed data access agreement with the authors.

References:

1. GBD 2017 Lower Respiratory Infections Collaborators. Quantifying risks and interventions that have affected the burden of lower respiratory infections among children younger than 5 years: an analysis for the Global Burden of Disease Study 2017. *Lancet Infect Dis* 2020;**20**:60-79.

2. Sonego M, Pellegrin MC, Becker G, Lazzerini M. Risk factors for mortality from acute lower respiratory infections (ALRI) in children under five years of age in low and middle-income countries: a systematic review and meta-analysis of observational studies. *PLoS One* 2015;**10**:e0116380.

Confidential: For Review Only

3. Lazzerini M, Sonego M, Pellegrin MC. Hypoxaemia as a Mortality Risk Factor in Acute Lower Respiratory Infections in Children in Low and Middle-Income Countries: Systematic Review and Meta-Analysis. *PLoS One* 2015;**10**:e0136166.

4. Theodoratou E, McAllister DA, Reed C, et al. Global, regional, and national estimates of pneumonia burden in HIV-infected children in 2010: a meta-analysis and modelling study. *Lancet Infect Dis* 2014;**14**:1250-8.

5. McCollum ED, Nambiar B, Deula R, et al. Impact of the 13-Valent Pneumococcal Conjugate Vaccine on Clinical and Hypoxemic Childhood Pneumonia over Three Years in Central Malawi: An Observational Study. *PLoS One* 2017;**12**:e0168209.

6. Gallagher KE, Knoll MD, Prosperi C, et al. The predictive performance of a pneumonia severity score in HIV-negative children presenting to hospital in seven low and middle-income countries. *Clin Infect Dis* 2020;**70**:1050–1057.

7. WHO. Integrated Management of Childhood Illness: Chart Booklet. 2014. Available: https://apps.who.int/iris/bitstream/handle/10665/104772/9789241506823_Chartbook_eng.pdf [Accessed 28 Dec 2021]

8. McCollum ED, King C, Hammitt LL, et al. Reduction of childhood pneumonia mortality in the Sustainable Development era. *Lancet Respir Med* 2016;**4**:932-933.

9. Smith AG, Eckerle M, Mvalo T, et al. CPAP IMPACT: a protocol for a randomised trial of bubble continuous positive airway pressure versus standard care for high-risk children with severe pneumonia using adaptive design methods. *BMJ Open Respir Res* 2017;**4**:e000195.

Confidential: For Review Only

10. McCollum ED, Mvalo T, Eckerle M, et al. Bubble continuous positive airway pressure for children with high-risk conditions and severe pneumonia in Malawi: an open label, randomised, controlled trial. *Lancet Respir Med* 2019;**7**:964-974.

11. WHO. Pocketbook of Hospital care for children. Guidelines for the management of common childhood illnesses. Second Edition, 2013. Available: https://apps.who.int/iris/bitstream/handle/10665/81170/9789241548373_eng.pdf?sequence=1 [Accessed 28 Dec 2021]

12. A Paediatric Handbook for Malawi. Third Edition, 2008. Available: http://cms.kcn.unima.mw:8002/moodle/file.php/1/Phillips_Kazembe_Paediatric_Handbook_for_Malawi_2008.pdf [Accessed 28 Dec 2021]

13. National Malaria Control Programme, Ministry of Health, Government of Malawi. Guidelines for the Treatment of Malaria in Malawi. 4th Edition, 2013. Available: https://www.severemalaria.org/sites/mmv-smo/files/content/attachments/2017-03-03/Malawi%202013%20Edition%20of%20Malaria%20Treatment%20Guidelines.pdf [Accessed 28 Dec 2021]

14. Ministry of Health. Malawi Standard Treatment Guidelines. 5th Edition, 2015. Available: https://extranet.who.int/ncdccs/Data/MWI_D1_Malawi-Standard-Treatment-Guidelines-Essential-Medicines-List-2015.pdf [Accessed 28 Dec 2021]

15. Liu L, Oza S, Hogan D, et al. Global, regional, and national causes of under-5 mortality in 2000-15: an updated systematic analysis with implications for the Sustainable Development Goals. *Lancet* 2016;**388**:3027-3035.

Confidential: For Review Only

16. Agweyu A, Lilford RJ, English M. Appropriateness of clinical severity classification of new WHO childhood pneumonia guidance: a multi-hospital, retrospective, cohort study. *Lancet Glob Health* 2018;**6**:e74-e83.

17. Berkley JA, Ross A, Mwangi I, et al. Prognostic indicators of early and late death in children admitted to district hospital in Kenya: cohort study. *BMJ*. 2003;**326**:361.

18. Graham H, Bakare AA, Ayede AI, et al. Diagnosis of pneumonia and malaria in Nigerian hospitals: A prospective cohort study. *Pediatr Pulmonol* 2020;**55** Suppl 1:S37-s50.

19. Sigaúque B, Roca A, Bassat Q, et al. Severe pneumonia in Mozambican young children: clinical and radiological characteristics and risk factors. *J Trop Pediatr* 2009;**55**:379-87.

20. Källander K, Nsungwa-Sabiiti J, Peterson S. Symptom overlap for malaria and pneumonia--policy implications for home management strategies. *Acta Trop* 2004;**90**:211-4.

21. Mwaniki MK, Nokes DJ, Ignas J, et al. Emergency triage assessment for hypoxaemia in neonates and young children in a Kenyan hospital: an observational study. *Bull World Health Organ* 2009;**87**:263-70.

22. Mogensen CB, Sørensen JE, Bjorkman A. Pallor as a sign of anaemia in small Tanzanian children at different health care levels. *Acta Trop* 2006;**99**:113-8.

23. Kalter HD, Burnham G, Kolstad PR, et al. Evaluation of clinical signs to diagnose anaemia in Uganda and Bangladesh, in areas with and without malaria. *Bull World Health Organ* 1997;**75** Suppl 1:103-11.

Confidential: For Review Only

24. Hooli S, King C, Zadutsa B, et al. The Epidemiology of Hypoxemic Pneumonia among Young Infants in Malawi. Am J Trop Med Hyg 2020;**102**:676-83.

25. Hooli S, Colbourn T, Lufesi N, et al. Predicting Hospitalised Paediatric Pneumonia Mortality Risk: An External Validation of RISC and mRISC, and Local Tool Development (RISC-Malawi) from Malawi. *PLoS One* 2016;**11**:e0168126.

26. Mayxay M, Pukrittayakamee S, Chotivanich K, Looareesuwan S, White NJ. Persistence of Plasmodium falciparum HRP-2 in successfully treated acute falciparum malaria. *Trans R Soc Trop Med Hyg* 2001;**95**:179-82.

Confidential: For Review Only

Table 1. Association between hospitalization characteristics and outcome among children 1-59 months old with WHO-defined severe pneumonia without HIV, severe malnutrition, and/or hypoxemia in an observational study

| Characteristic | | Survived N=863 | Died N=21 | p-value |
|---|---|---|---|---|
| Demographics | | | | |
| Age in months, median (IQR)[1] | | 9.2 (4.7, 17.3) | 8.1 (6.5, 12.0) | 0.74 |
| Sex, n (%)[2] | Females | 353 (97.5%) | 9 (2.5%) | 1.00 |
| | Males | 510 (97.7%) | 12 (2.3%) | |
| Clinical features | | | | |
| Respiratory rate (breaths/min), mean (SD)[3] | | 59.6 (12.9) | 60.3 (13.7) | 0.82 |
| Fast breathing for age, n (%)[2] | Yes | 720 (97.6%) | 18 (2.4%) | 1.00 |
| | No | 143 (97.9%) | 3 (2.1%) | |
| Pulse rate (beats/min), mean (SD)[3] | | 162.2 (20.4) | 170.0 (30.4) | 0.09 |
| Pulse rate >180 beats/min, n (%)[2] | Yes | 113 (95.0%) | 6 (5.0%) | 0.22 |
| | No | 400 (97.6%) | 10 (2.4%) | |
| Pulse rate >160 beats/min, n (%)[2] | Yes | 165 (97.6%) | 4 (2.4%) | 0.20 |
| | No | 184 (99.5%) | 1 (0.5%) | |
| Severe chest indrawing, n (%)[2] | Yes | 760 (97.8%) | 17 (2.2%) | 0.31 |
| | No | 103 (96.3%) | 4 (3.7%) | |
| Head nodding, n (%)[2] | Yes | 180 (95.7%) | 8 (4.3%) | 0.10 |
| | No | 683 (98.1%) | 13 (1.9%) | |
| Grunting, n (%)[2, 5] | Yes | 102 (91.1%) | 10 (8.9%) | <0.01 |
| | No | 760 (98.6%) | 11 (1.4%) | |
| Stridor when calm, n (%)[2] | Yes | 12 (85.7%) | 2 (14.3%) | 0.04 |
| | No | 851 (97.8%) | 19 (2.2%) | |
| Apnea, n (%)[2] | Yes | 6 (85.7%) | 1 (14.3%) | 0.16 |
| | No | 857 (97.7%) | 20 (2.3%) | |
| Nasal flaring, n (%)[2] | Yes | 507 (97.6%) | 12 (2.3%) | 1.00 |
| | No | 356 (97.5%) | 9 (2.5%) | |
| Measurements and laboratory results | | | | |
| MUAC in cm, mean (SD)[3] | | 14.0 (1.2) | 13.3 (1.0) | <0.01 |
| MUAC 11.5-13.5 cm, n (%)[2] | Yes | 255 (96.2%) | 10 (3.8%) | 0.09 |
| | No | 608 (98.2%) | 11 (1.8%) | |
| Hb (g/dL), mean (SD)[3] | | 10.0 (2.0) | 7.8 (3.0) | <0.01 |
| Hb <5 g/dL[2] | Yes | 24 (88.9%) | 3 (11.1%) | 0.02 |
| | No | 839 (99.6%) | 18 (2.%) | |
| Malaria positive, n (%)[4] | Yes | 193 (94.6%) | 11 (5.4%) | <0.01 |
| | No | 663 (98.5%) | 10 (1.5%) | |

Confidential: For Review Only

| | | | | |
|---|---|---|---|---|
| $SpO_2$ (%), median (IQR)[1] | | 96 (95, 98) | 96 (94, 98) | 0.49 |
| $SpO_2$[2] | 93-100% | 787 (97.8%) | 18 (2.2%) | 0.42 |
| | 90-92% | 76 (96.2%) | 3 (3.7%) | |

WHO indicates World Health Organization; IQR, interquartile range; SD, standard deviation; MUAC, mid-upper arm circumference; Hb, hemoglobin.

[1]Wilcoxon rank sum

[2]Fisher's exact

[3]Two sample t-test

[4]7 participants were missing test results

[5]1 participant missing data

Confidential: For Review Only

463 Table 2: Association between mortality and number of risk factors (stridor when calm,
464 grunting, hemoglobin <5 g/dL, malaria test positive)
465

| Number of risk factors | Odds ratio (95% CI) | p-value |
|---|---|---|
| 1 | Referent | – |
| 2 | 4.5 (1.4, 14.1) | <0.01 |
| 3 | 14.7 (4.3, 50.1) | <0.01 |
| 4 | 26.2 (4.4, 153.7) | <0.01 |

466

Confidential: For Review Only

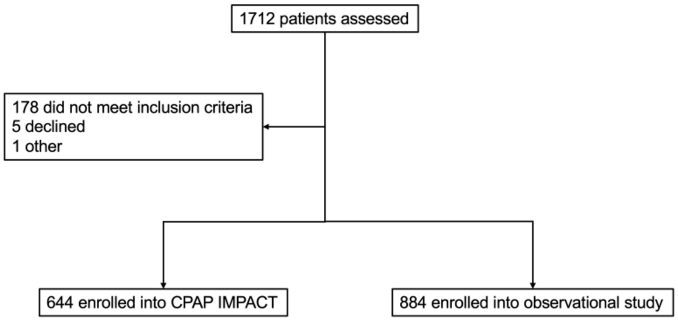


1649x1165mm (72 x 72 DPI)

Supplemental Materials

Supplemental Table 1: Sensitivity analysis of survival and death, excluding children with no malaria result

| Factor | | Survived | Died | p-value |
|---|---|---|---|---|
| N=877 | | 856 | 21 | |
| Sex, n (%) | Males | 505 (59.0%) | 12 (57.1%) | 1.00 |
| | Females | 351 (41.0%) | 9 (42.9%) | |
| Age in months, median (IQR) | | 9.2 (4.6, 17.2) | 8.1 (6.5, 12.0) | 0.76 |
| Respiratory rate (breaths/min), mean (SD) | | 59.6 (12.9) | 60.3 (13.7) | 0.82 |
| Fast breathing for age | No | 143 (16.7%) | 3 (14.3%) | 1.00 |
| | Yes | 713 (83.3%) | 18 (85.7%) | |
| Pulse rate (beats/min), mean (SD) | | 162.3 (20.4) | 170.0 (30.4) | 0.09 |
| Pulse rate >180 beats/min, n (%) | No | 397 (77.8%) | 10 (62.5%) | 0.22 |
| | Yes | 113 (22.2%) | 6 (37.5%) | |
| Pulse rate >160 beats/min, n (%) | No | 181 (52.5%) | 1 (20.0%) | 0.20 |
| | Yes | 164 (47.5%) | 4 (80.0%) | |
| Severe chest indrawing, n (%) | No | 99 (11.6%) | 4 (19.0%) | 0.30 |
| | Yes | 757 (88.4%) | 17 (81.0%) | |
| Head nodding, n (%) | No | 676 (79.0%) | 13 (61.9%) | 0.10 |
| | Yes | 180 (21.0%) | 8 (38.1%) | |
| Grunting, n (%) | No | 754 (88.2%) | 11 (52.4%) | <0.01 |
| | Yes | 101 (11.8%) | 10 (47.6%) | |
| Stridor when calm, n (%) | No | 844 (98.6%) | 19 (90.5%) | 0.04 |
| | Yes | 12 (1.4%) | 2 (9.5%) | |
| Apnea, n (%) | No | 850 (99.3%) | 20 (95.2%) | 0.16 |
| | Yes | 6 (0.7%) | 1 (4.8%) | |
| Nasal flaring, n (%) | No | 350 (40.9%) | 9 (42.9%) | 1.00 |
| | Yes | 506 (59.1%) | 12 (57.1%) | |
| MUAC in cm, mean (SD) | | 14.0 (1.2) | 13.3 (1.0) | <0.01 |
| MUAC 11.5-13.5 cm, n (%) | No | 604 (70.6%) | 11 (52.4%) | 0.09 |
| | Yes | 252 (29.4%) | 10 (47.6%) | |
| Hb (g/dL), mean (SD) | | 10.0 (2.0) | 7.8 (3.0) | <0.01 |
| Hb < 5 g/dL | No | 832 (97.2%) | 18 (85.7%) | 0.02 |
| | Yes | 24 (2.8%) | 3 (14.3%) | |
| SpO2 (%), median (IQR) | | 96.0 (95.0, 98.0) | 96.0 (94.0, 98.0) | 0.49 |

Confidential: For Review Only

| | | | | |
|---|---|---|---|---|
| SpO2 93-100% | 0 | 781 (91.2%) | 18 (85.7%) | 0.42 |
| SpO2 90-92% | 1 | 75 (8.8%) | 3 (14.3%) | |
| Malaria positive, n (%) | No | 663 (77.5%) | 10 (47.6%) | 0.003 |
| | Yes | 193 (22.5%) | 11 (52.4%) | |

Confidential: For Review Only

Supplemental Table 2. Characteristics of children 1-59 months old with WHO-defined severe pneumonia at Salima District Hospital, Malawi in an observational study compared to a randomized controlled trial CPAP IMPACT

| Characteristic | Observational study N=884 | CPAP IMPACT N=644 | p-value |
|---|---|---|---|
| Age in months, median (IQR) | 9.2 (4.7, 17.2) | 7.7 (3.1, 15.2) | <0.01 |
| Females, n (%) | 362 (41.0%) | 299 (46.4%) | 0.03 |
| Males, n (%) | 522 (59.0%) | 345 (53.6%) | |
| Weight in kg, mean (SD) | 8.5 (2.5) | 7.2 (2.8) | <0.01 |
| MUAC in cm, mean (SD) | 14.0 (1.2) | 12.7 (1.9) | <0.01 |
| HIV-infected, n (%) | 0 (0.0%) | 36 (5.6%) | <0.01 |
| HIV-exposed uninfected, n (%) | 0 (0.0%) | 99 (15.4%) | <0.01 |
| Severe malnutrition, n (%) | 0 (0.0%) | 222 (34.5%) | <0.01 |
| Hypoxemia only, n (%) | 0 (0.0%) | 287 (44.6%) | <0.01 |
| Pulse rate in beats/min, mean (SD) | 162.4 (20.7) | 164.1 (24.0) | 0.15 |
| Respiratory rate in breaths/min, mean (SD) | 59.6 (12.9) | 61.9 (15.1) | <0.01 |
| $SpO_2$, median (IQR) | 96 (95, 98) | 88 (83, 94) | <0.01 |
| $SpO_2$ 93-100%, n (%) | 805 (91.1%) | 191 (29.7%) | |
| $SpO_2$ 90-92%, n (%) | 79 (8.9%) | 38 (5.9%) | |
| $SpO_2$ <90%, n (%) | 0 (0.0%) | 415 (64.4%) | |
| 1 or more respiratory danger signs[1], n (%) | 866 (98.0%) | 638 (99.1%) | 0.09 |
| Severe chest indrawing, n (%) | 777 (87.9%) | 575 (89.3%) | 0.41 |
| Head nodding, n (%) | 188 (21.3%) | 176 (27.3%) | <0.01 |
| Grunting, n (%) | 112 (12.7%) | 154 (23.9%) | <0.01 |
| Very fast breathing for age, n (%)[2] | 256 (29.0%) | 211 (32.8%) | 0.11 |
| Stridor when calm, n (%) | 14 (1.6%) | 13 (2.0%) | 0.52 |
| Apnea, n (%) | 7 (0.8%) | 37 (5.7%) | <0.01 |
| Nasal flaring, n (%) | 519 (58.7%) | 471 (73.1%) | <0.01 |
| Convulsions, n (%) | 45 (5.1%) | 61 (9.5%) | <0.01 |
| Inability to feed, n (%) | 27 (3.1%) | 78 (12.1%) | <0.01 |
| Vomiting everything, n (%) | 12 (1.4%) | 19 (3.0%) | 0.03 |
| Blantyre Coma Score ≤4, n (%) | 20 (2.3%) | 55 (8.5%) | <0.01 |
| Crackles on auscultation, n (%) | 429 (48.5%) | 311 (48.3%) | 0.95 |
| Wheeze on auscultation, n (%) | 160 (18.1%) | 128 (19.9%) | 0.38 |
| Hb <5 g/dL, n (%) | 27 (3.1%) | 50 (7.8%) | <0.01 |
| Hb (g/dL), mean (SD) | 10.0 (2.1) | 9.5 (2.6) | <0.01 |
| Malaria positive, n (%)[3] | 204 (23.3%) | 197 (30.6%) | <0.01 |
| Death, n (%) | 21 (2.4%) | 88 (13.7%) | <0.01 |

WHO indicates World Health Organization; IQR, interquartile range; SD, standard deviation; MUAC, mid-upper arm circumference; $SpO_2$; peripheral oxyhemoglobin saturation; Hb, hemoglobin.

[1]Any of the following signs of respiratory distress: severe chest indrawing, head nodding, grunting, very fast breathing for age, stridor when calm, apnea, nasal flaring

Confidential: For Review Only

[2]≥80 breaths/min if 30-59 days old, ≥70 breaths/min if 2-11 months old, and ≥60 breaths/min if 12-59 months old

[3]Rapid test positive, 10 observational study participants were missing test results

Confidential: For Review Only
